# Population estimates of Bornean orang-utans using Bayesian analysis at the greater Batang Ai-Lanjak-Entimau landscape in Sarawak, Malaysia

**DOI:** 10.1038/s41598-018-33872-3

**Published:** 2018-10-23

**Authors:** Joshua Pandong, Melvin Gumal, Lukmann Alen, Ailyn Sidu, Sylvia Ng, Lian Pin Koh

**Affiliations:** 1 Wildlife Conservation Society (WCS)-Malaysia Programme, No. 7 Jalan Ridgeway, 93200 Kuching, Sarawak Malaysia; 2 Present Address: School of Biological Sciences, The University of Adelaide, South Australia, 5005 Australia; 3 WWF Malaysia, Bangunan Binamas 7th Floor, Jalan Padungan, Kuching, Sarawak Malaysia; 4 Conservation International, 3131 East Madison Street, Suite 201, Seattle, WA 98112 USA

## Abstract

The integration of Bayesian analysis into existing great ape survey methods could be used to generate precise and reliable population estimates of Bornean orang-utans. We used the Marked Nest Count (MNC) method to count new orang-utan nests at seven previously undocumented study sites in Sarawak, Malaysia. Our survey teams marked new nests on the first survey and revisited the plots on two more occasions; after about 21 and 42 days respectively. We used the *N*-mixture models to integrate suitability, abundance and detection models which account for zero inflation and imperfect detection for the analysis. The result was a combined estimate of 355 orang-utans with the 95% highest density interval (HDI) of 135 to 602 individuals. We visually inspected the posterior distributions of our parameters and compared precisions between study sites. We subsequently assess the strength or reliability of the generated estimates using identifiability tests. Only three out of the seven estimates had <35% overlap to indicate strong reliability. We discussed the limitations and advantages of our study design, and made recommendations to improve the sampling scheme. Over the course of this research, two of the study sites were gazetted as extensions to the Lanjak-Entimau Wildlife Sanctuary for orang-utan conservation.

## Introduction

The world’s three orang-utan species, Sumatran (*Pongo abelii*), Bornean (*Pongo pygmaeus*) and Tapanuli orang-utans (*Pongo tapanuliensis*) are listed as Critically Endangered under the IUCN Red List of Threatened Species, with the latter described and listed in 2017^1–3^. Threats to orang-utan survival have intensified in the past 60 years due to rapid deforestation^4,5^, land use conversion into monoculture plantations^6,7^, habitat fragmentation^8^, illegal wildlife trade and hunting of the species^9,10^. The Bornean orang-utan populations suffered more than 25% decline between 1997 and 2015^11,12^ despite an increase in scientific interest and public support. The decline is likely to continue in the immediate future considering social and economic circumstances^13^ and the economic importance of oil palm plantations in Malaysia and Indonesia^14^.

Due to limited data collection, continued monitoring of orang-utan abundance is crucial to assess their population status and rates of population decline^12,15^. This is in line with implementing the Orang-utan Population and Habitat Viability Assessment (PHVA) mitigation measures with the goals of maintaining high forest cover at orang-utan habitats and improving connectivity between forest patches with orang-utans^11,12^. However, it is rarely feasible to acquire accurate population and density estimates from direct counts of orang-utans in the wild. The great apes are elusive, solitary and live in small population sizes which require greater effort to detect^13,16^.

Researchers in general opted for indirect sign counts to generate population estimates due to constraints on direct counts^16,17^. For orang-utans, this means counting nests instead of individuals or groups. The advantages of using nest counts include: (a) nests are proxies for orang-utans; (b) indicator of active habitat use as weaned individuals build nests on an almost daily basis to sleep at night or sometimes to rest during the day; (c) higher encounter rates than encounters with great apes; and (d) easier measurement of perpendicular distances as nests are stationary^16,17^.

Currently, the standard survey protocol to estimate orang-utan density consists of: (a) counting all nests visible from a line transect or plot; (b) generating nest density within the area surveyed; and (c) converting nest density into orang-utan density using an algorithm^16,18^. There are two methods to generate orang-utan density using various parameters: (1) the standing crop nest count (SCNC) method uses nest decay rate, nest construction rate and the records of all nests encountered; and (2) the marked nest count (MNC) method uses only nest construction rate and the records of new nests built within a known inter-survey period^16,18–20^.

Both the SCNC and MNC methods are being used for long-term nest monitoring in Borneo and Sumatra^18,21,22^. Although, these methods have limitations, Marshall & Meijaard^23^ warned that the nest decay rate in SCNC is the most problematic source of error when used to estimate orang-utan density. It is often based on nest decay rates from other sites and time periods when an empirical rate is not available for a particular study region. This approach adds much uncertainty and error to the population estimation because nest decay rates are affected by environmental and biophysical conditions, which vary across space and time^16,24–26^.

To bypass nest decay rate, the MNC method uses records of only newly-built nests between the first and the last survey^19,20^. MNC assumes that all new nests were marked and recorded on the first survey, and no new nests built and decayed between the inter-survey periods. However, despite bypassing nest decay rate, Spehar *et al*.^18^ found that the time and effort to acquire reliable and precise density estimates were reportedly the same as the SCNC method. Due to low number of new nests found, past MNC studies of great apes by Plumptre *et al*.^20^ and Spehar *et al*.^18^ recommended a sample size of 50 new nests and survey effort of more than 200 km of line transects to be sufficient.

Distance sampling is the most widely used technique to analyse line transect data at present^16,27^. The technique uses detection functions to model the probability of nest detection given the perpendicular distance (or shortest distance) between the observer and the nest. It is expected that the probability of nest detection rapidly decreases with increasing distance from the observer. Nest density estimate is subsequently generated by combining the model with nest encounter rate at the study site. However, the minimum sample size of 60 nests applies to acquire a precise estimate^27^. It is possible to pool nest data from all months to obtain an overall orang-utan density estimate for study site with low nest detections^18^. But this may result in imprecise estimates with wide confidence intervals.

In this paper, we show how the integration of the Bayesian framework into the analysis of density estimates is a novel approach. We applied the *N*-mixture models to simultaneously model suitability, abundance and detection^28^. For the surveys, we opted to use the MNC method and plot survey, instead of the standard line transect (Supplementary Table S1). The suitability model relates to whether or not a plot has old or new orang-utan nest, which is an indicator of active habitat use; whilst the abundance model refers to new nests abundance given the suitability model. We were able to quantify and visually inspect the most credible range of possibilities and covered 95% of the probability distribution as the highest density interval (HDI)^29^. Finally, we ran identifiability tests to assess the strength or reliability of our estimates^30^.

Given the above, the aims of this paper are to: (a) integrate the Bayesian analysis into the MNC method to generate density and population estimates; and (b) assess the strength or reliability of these estimates. We conducted nest count surveys of Bornean orang-utans (subspecies *Pongo pygmaeus pygmaeus*) at seven previously undocumented study sites in the Malaysian state of Sarawak. We also compared the results with a non-Bayesian approach, discussed the limitation and advantages of using the Bayesian analysis, and the conservation implications of our paper.

## Results

### Population and density estimates of orang-utans at the study sites

**Figure 1:**
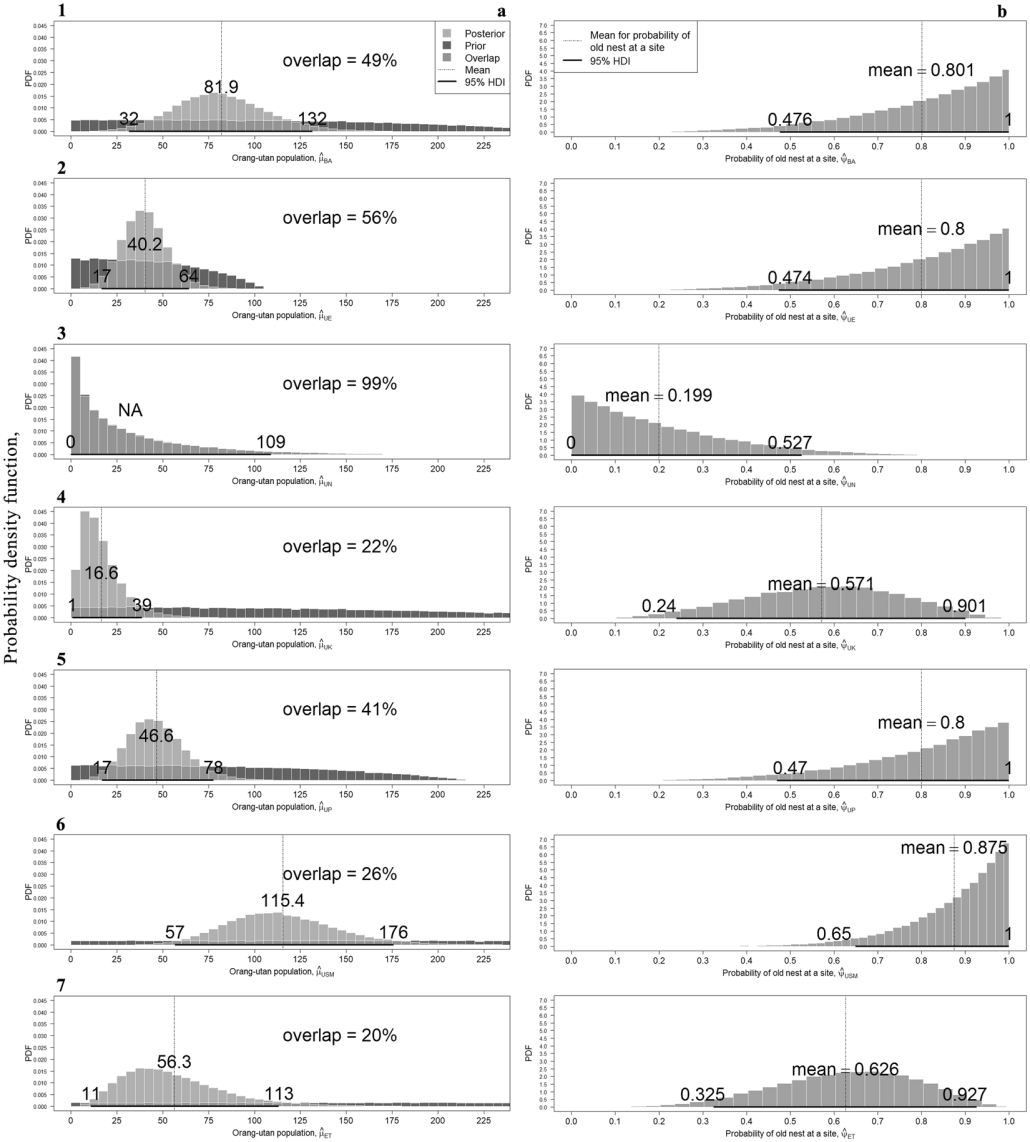
The columns correspond to the probability density function of: (**a**) prior and posterior estimates of orang-utan population, and (**b**) probability of old nest at a site with 95% HDI at the seven study sites. The row sequence corresponds to the study sites: (1) Batang Ai, (2) Ulu Engkari, (3) Ulu Ngemah, (4) Ulu Katibas, (5) Ulu Pasin, (6) Ulu Sungai Menyang, and (7) Engkari-Telaus.

The combined estimate of orang-utan population ($\hat{\mu }$) at the seven study sites was 355 individuals with 135 to 602 individuals within the 95% highest density interval (HDI). The combined orang-utan density ($\hat{d}$) was 0.5249 individuals/km^2^ with 0.1964 to 0.8842 individuals/km^2^ 95% HDI (Table 1 and Fig. 1).

**Table 1:** Estimates of probability of detecting new nest ($\hat{q}$), new orang-utan nests recorded on the first survey (${\hat{x}}_{0}$), orang-utan density, $\hat{d}$ (orang-utans km^−2^), and orang-utan population ($\hat{\mu }$) with 95% HDI for the study sites.

Study site	Acronym	$\hat{q}$ (95% HDI)	${\hat{x}}_{0}$ (95% HDI)	Estimate of orang-utan density			Estimate of orang-utan population		
				$\hat{d}$	Lower HDI	Upper HDI	$\hat{\mu }$	Lower HDI	Upper HDI
1. Southern (Batang Ai)	BA	0.8133 (0.6586 to 0.9412)	43.4237 (36.0003 to 52.6110)	1.4050	0.5440	2.2586	**82**	**32**	**132**
2. Northern (Ulu Engkari)	UE			1.7890	0.7442	2.8663	**40**	**17**	**64**
3. Ulu Ngemah	UN			0.4681	0.0000	1.5696	**NA**	**NA**	**NA**
4. Ulu Katibas	UK			0.1719	0.0080	0.4014	**16**	**1**	**39**
5. Ulu Pasin	UP			1.0174	0.3682	1.6944	**46**	**17**	**78**
6. Ulu Sungai Menyang	USM			0.8245	0.4052	1.2554	**115**	**57**	**176**
7. Engkari Telaus	ET			0.2273	0.0434	0.4571	**56**	**11**	**113**
Total combined estimate with 95% HDI at the study sites:				0.5249*	0.1964*	0.8842*	**355**	**135**	**602**

We do not have an estimate of orang-utan population at Ulu Ngemah given that there was no evidence of habitat use by orang-utans during the survey duration. We assigned ‘NA’ for the estimates. *Note: The density estimates here were weighted by the area size of the study sites.

### Precision and reliability of the estimates

The precision of an estimate is visually inspected by the shape of the histogram. For our study sites, the population estimate at Ulu Katibas had the highest precision. This is visible as the distribution with the narrowest 95% HDI, and the highest and sharpest peak of the seven sites (Fig. 1). The distribution at Ulu Sungai Menyang shows that the estimate was less precise with wider 95% HDI and flatter peak than Ulu Katibas, even though it had the highest mean of 115 orang-utans. The shape for Ulu Ngemah is notably skewed to the right with the highest peak (mode or the most probably value) at zero. This is the only study site where we were not able to generate an estimate based on the data we collected.

Gimenez *et al*.^30^ suggested an identifiability test to assess the strength of an estimate with a guideline that 35% overlap or more between the posterior and prior distributions was ‘an indicator of weak identifiability of a parameter’. Out of the seven study sites, the posterior distributions for Batang Ai, Ulu Engkari, Ulu Pasin and Ulu Ngemah had an overlap of more than 35%, which indicated that our orang-utan population estimates ($\hat{\mu }$) for these sites were weak. In contrast, the posterior distributions for Ulu Katibas, Ulu Sungai Menyang and Engkari-Telaus were clearly different with overlaps of 22%, 26% and 20% respectively. Thus, our estimates of $\hat{\mu }$ at these sites were strongly reliable and estimable (Fig. 1).

Results from a non-Bayesian approach for our datasets show that two of the seven sites had problematic estimates due to low number of new nests detected (Supplementary Figs S1–S3). The problematic estimates generated via the bootstrapping analysis were: (a) 7 individuals with 95% confidence interval (CI) of 0 to 17 individuals for Ulu Katibas; and (b) 37 individuals with 95% CI of 0 to 85 individuals for Engkari-Telaus. The lower limit of 95% CI for (a) and (b) should include at least one orang-utan given new nests were recorded during the inter-survey period. The inclusion of zero orang-utan at the lower limit was due to a large proportion of zero values computed from the bootstrapping analysis^31^.

### Other parameters of interest

A total of 29 plots were surveyed with a combined plot area size of 4.27 km^2^ or 0.63% of the combined study area (680.21 km^2^) (Supplementary Table S2, and Supplementary Figs S4 and S5). The average plot size for the 29 plots surveyed was 0.1471 km^2^, or four 1-km strips per plot with strip width of 36 m (Supplementary Figs S6 and S7). There were 20 plots revisited on the second and third surveys with an average of 42.7 days between the first and the third survey. Two plots were visited on the second survey but not revisited on the third due to logistics constraint. Seven plots were not revisited on the second and third surveys as no old or new orang-utan nest detected in these plots on the first survey.

We used detection of old or new orang-utan nest on the first survey ($\hat{\psi }$) as an indicator of habitat use by orang-utans at the plots. There were four left skewed distributions for $\hat{\psi }$ with the highest peaks at 1 (Fig. 1). Given our data, this was an indication of 100% habitat use by the orang-utans present at all plots in Batang Ai, Ulu Engkari, Ulu Pasin and Ulu Sungai Menyang during the survey duration. The highest mean for $\hat{\psi }$ at 87.5% in Ulu Sungai Menyang landscape shows the importance of this non-protected landscape for long-term orang-utan conservation. In contrast, the distribution at Ulu Ngemah was right skewed and had the highest peak at zero. But we did not assume habitat use was 0% at the plots since the result in Ulu Ngemah was unestimable given our data during the survey duration. For Ulu Katibas and Engkari-Telaus, not all plots were used by orang-utans at the two study sites as shown by the 95% HDI spread around values less than 1 for $\hat{\psi }$ distributions.

There were 40 new orang-utan nests recorded by the two teams combined during the first surveys (*x*) at 29 plots; whilst the total number of new nests recorded on the second and third surveys (*y*) was 93 new nests (Supplementary Table S2). We assessed the estimated probability of detecting new nest by two teams on the first survey ($\hat{q}$). Team 1 missed five nests in total but were recorded by Team 2 from the opposite direction. The estimate of $\hat{q}$ for all the study sites was 0.8133 within 95% HDI of 0.6586 to 0.9412 (Table 1). This estimate was used for the detection of new nests in subsequent visits (second and third surveys). As 1.0000 was not within the 95% HDI, not all new orang-utan nests were detected even if they were present at the plots.

Given the data, it was also possible to estimate the overall new orang-utan nests built during the first surveys at the seven study sites (${\hat{x}}_{0}$). Although 40 new orang-utan nests were recorded, the estimate of ${\hat{x}}_{0}$ was 43 new orang-utan nests within 95% HDI of 36 to 52 nests during the first surveys (Table 1). For further information on the data collected and additional results, refer to the Supplementary Appendix S1.

## Discussion

Our results show that integrating the Bayesian analysis into the MNC method allowed us to generate more precise estimates even with low counts of new nest. However, we were only able to generate reliable estimates for three of the seven study sites due to insufficient number of plots surveyed in the other four. We acknowledge that time and survey effort invested in the MNC method of this paper was likely the same as the SCNC method from previous studies in Borneo by Spehar *et al*.^18^ and van Schaik *et al*.^21^. But we compensate this by quantifying and visually inspecting the precision for all our parameters of interest, in contrast with the uncertainty in nest decay estimation using the SCNC method. In this section, we further discuss the two components of the *N*-mixture models, the limitation and advantages of using the Bayesian analysis in the MNC method, as well as the conservation implications of our paper on the study sites.

### Zero inflation and imperfect detection in the *N*-mixture models

One of the initial concerns for the MNC method was the low counts of new nest observed, and not meeting the recommended sample size of 50 new nests and survey effort of more than 200 km. We initially ran the bootstrapping analysis for plots without any new nests (or in very small numbers) but had signs of habitat use by orang-utans. Some of these results were indeed problematic with population estimates ranging from zero orang-utan and did not fit the standard distributions due to high number of bootstrap samples computed containing the value zero. We then compared this non-Bayesian bootstrapping approach with a Bayesian framework that accounts for subjective belief about our study sites. The first step in our *N*-mixture modelling was then to identify the source of zero inflation and use the models to examine the ecological process.

We adapted Martin *et al*.’ s^31,32^ descriptions into our context for the two types of zero values that vary in four ways. The first type is ‘true zero’ and it refers to: (a) zeros due to random local orang-utan extinction at suitable habitats; and (b) zeros due to strong ecological processes such as unsuitable habitat from disturbances or poor vegetation quality. The second type is ‘false zero’ and it refers to: (c) zeros from temporary absence due to large home ranges; and (d) zeros due to imperfect detection by observers due to the detection process and variability of new nests sampled. To account for definitions (b) and (d), we combined the zero-inflated Poisson to model new nest abundance given a Bernoulli distribution for site suitability based on plots used or not used ($\hat{\psi }$) as the latent process. The number of new nests recorded (*y*) was then based on a Binomial distribution to account for imperfect detection ($\hat{q}$) as the observation process^33,34^.

However, we made no inferences about true zeros due to local extinction at suitable habitats nor about false zeros due to temporary orang-utan absences relative to large home ranges. This is of concern in view that we did not observe any new or old orang-utan nest at Ulu Ngemah at all three plots, and did not revisit for the second and third surveys. We made the decision based on Assumption #3 of our methodology as we found no evidence that orang-utans were present. Thus, the most appropriate inference for Ulu Ngemah is to say we were 95% certain that habitat use by orang-utans at the plots surveyed was less than 5%.

To reiterate, estimates generated via the MNC method in this paper represent only orang-utans present at the plots during the survey period, which is an average of 42.7 days between the first and the last survey. The estimates based on our data were site-specific, reflected a snapshot at a given point in time and did not reflect an overall steady population density at the study sites^18^. Wich & van Schaik^35^ noted that orang-utans do range extensively across habitats and their abundance in certain areas may correlate to wild fruit abundance at the time. The inference of our estimates then were made after deliberating only habitat use by orang-utans within surveyed plots, over a short survey period, and with imperfect detection by observers.

### Limitation and advantages of using the Bayesian analysis

Having prior credibility is a critical part of Bayes’ Rule^29^. But up to this point, we did not have previous knowledge of orang-utan numbers at the study sites. Hence, we opted for an uninformative prior (or flat prior) as our prior credibility. This became problematic for Ulu Ngemah as no old or new nest was recorded at the study site. The posterior distribution for $\hat{\mu }$ then spread widely within 95% HDI of 0 to 109 individuals and had 99% overlap with the uninformative prior, which can be misleading. A more sensible approach to estimate parameter of interest when none of the plots had new nests is to use (or borrow) estimates from nearby study sites as informative priors. The strength and reliability assessment can then be used to measure the posterior-prior overlap percentages to justify the results.

There were three assumptions in this study that determined key decisions when conducting our survey methodology. These assumptions were adapted from the standard MNC method and distance sampling theory^16,27^. All three assumptions were violated at some point, and were either resolved during subsequent surveys, quantified for a measure of precision, or amended for future surveys. Violation of these assumptions meant that we detected less new nests which may have led to an underestimation of nest construction rate, orang-utan density and population estimates generated in this study.

Assumption #1 states that ‘all orang-utan nests present at the plot were recorded on the first survey’. This is adapted from one of the critical assumptions underlying the distance sampling theory except that distance sampling uses line or point transects. Our survey design allowed this assumption to be assessed by having two teams searching each strip for new nests on the same day. There were at least five new nests missed by Team 1 but were later detected by Team 2 from the opposite direction. The assessment result indeed showed probability of detecting new nests ($\hat{q}$) to be <1, which was at 81.33%, 95% HDI of 65.86% to 94.12%. This shows that not all nests present at the plots were recorded by the two teams.

Assumption #2 states that ‘no nest will be constructed and then visible as old before the next survey (21 days later)’^16,19,20^. On at least three occasions, Class C nests were detected by the teams on subsequent surveys. This could mean that nests were built after the first survey and decayed before the second survey, or the nests were missed on the first survey entirely. Another possible situation that violates this assumption was to have new nests built on the first survey but missed by both teams, and was still new when it was detected during the subsequent survey. We addressed this possible violation by conducting one-day training on at least five occasions for inexperienced field assistants and researchers on detecting and observing different nests classes. In addition, for future surveys, we may opt for a shorter inter-survey period of 14 to 21 days^20^ to avoid nest decaying before the next survey.

Assumption #3 was the most challenging as we assumed that ‘plots with no orang-utan nests (old/new) at the first survey were assumed not to be used in the next 42 days (one survey duration) by orang-utans, and nest construction rate was zero’. The main reason for this assumption was initially as a cost effective measure. Our study then does not infer whether unused plots were due to local extinction, temporary absence or a non-habitat for orang-utans. But we highly recommend the application of our study design as possible follow up studies in the area, albeit with more plots, more revisits and a different set of research questions.

It is a great advantage for the Bayesian framework in our *N*-mixture models to allow even low counts of new nests to generate precise estimates. However, sufficient number of plots and revisits are prerequisites to generate reliable estimates. For our results, the estimate at Ulu Katibas was the most precise and the second most reliable of the seven study sites despite having only two new nests recorded on the second and third surveys. This was due to five plots surveyed in contrast to only three plots at four study sites with weak estimates. Although the *N*-mixture models do accommodate zero counts, Dénes *et al*.^33^ cautioned that the analysis would induce errors if estimates were generated at habitats where the species does not occur. Therefore, previous knowledge of orang-utan habitats is also crucial to ensure a precise and reliable estimate at the study site with low or zero counts of new nest.

We believe there is potential to expand the use of MNC and the Bayesian analysis into existing, alternative and/or integrative methods to study great ape population sizes. It is also possible to adapt this method to account for indices of animal density based on sign density such as dung piles or tracks. The end goal of using the MNC method is not just to acquire current density and population estimates given that the method is appropriate for short term studies. But there is also potential to integrate with spatial data to map long term great ape movement, population viability, and seasonal habitat use within a study site^14,18^. Santika *et al*.^11^ already applied the Bayesian analysis in an integrated analysis to estimate the rate and drivers of orang-utan decline in Borneo. The identifiability tests by Gimenez *et al*.^30^ can also take similar analysis a step further by assessing the strength or reliability of the estimates.

### Conservation implications on the study sites

The results of this study provided updates on the remaining orang-utan populations in Sarawak. This is critical to help develop more effective and proactive conservation strategies for the species. The last reliable estimate of orang-utan abundance in Sarawak was published more than two decades ago^36,37^, although there have been ad hoc field surveys conducted between 2003 and 2007^38^. However, the data for the 2003–2007 surveys was not published in a peer-review document as the estimates were deemed inappropriate due to an issue of bias (surveys were conducted only on ridges, following those conducted in 1993)^36^. Nevertheless internal and donor reports were generated for the surveys conducted at the core orang-utan habitats of Batang Ai-Lanjak-Entimau (BALE) landscape. The reports provided an estimate of 1,984 orang-utans with 95% CI of 1,175 to 2,582 individuals. However, this estimate is credible only if the whole BALE was homogenous and consisted of ridges.

In 2013, two study sites namely Ulu Katibas and Ulu Pasin were gazetted as extensions to the Lanjak-Entimau Wildlife Sanctuary or LEWS. The data generated from these surveys were instrumental in justifying the creation of the new protected areas. Active habitat use by orang-utans at the study sites confirmed their status as high conservation value forests despite the weak estimate at Ulu Pasin and low estimate at Ulu Katibas. The joint partners of this study also proposed a list of prescribed activities including the prohibition of large-scale land use conversion for commercial purposes for the non-protected landscapes of Ulu Sungai Menyang and Engkari-Telaus. This is in line with the Sarawak Government’s policy of zero loss of orang-utans and their landscapes since 2016.

## Conclusion

In this paper, we managed to generate estimates of orang-utan density and population even with low counts of new nests. We addressed the concerns of previous studies that required a minimum number of 50 new nests before a precise estimate can be generated. This was resolved by using *N*-mixture models to account for zero-inflation and imperfect detection. The estimated total population for the combined seven previously undocumented study sites was 355 orang-utans with 95% HDI of 135 to 602 individuals. However, we found that four out of the seven estimates to be weak and misleading after performing the identifiability tests. The reasons for the weak estimates were due to: (a) insufficient plots surveyed at those sites; (b) possible violations of the three main assumptions leading to an underestimation of new nests detected; and (c) the use of an uninformative prior which became problematic as the posterior distribution had a 99% overlap with its prior distribution, rendering our output not estimable. For future replication of our sampling scheme, we recommend: (1) at least six or more plots surveyed and revisited depending on the simulated outcome of having <35% overlap in an identifiability test; (2) a shorter inter-survey period of 14 to 21 days; and (3) the use of an informative prior with strong reliability from nearby sites to generate more sensible estimates for study sites without old or new orang-utan nest detected. The concept for the Bayesian framework and identifiability test of this paper allowed stronger and reliable estimates to be generated with a measure of precision, and it is applicable to existing, alternative and integrative methods for orang-utan studies.

## Methods

### Study area

**Figure 2:**
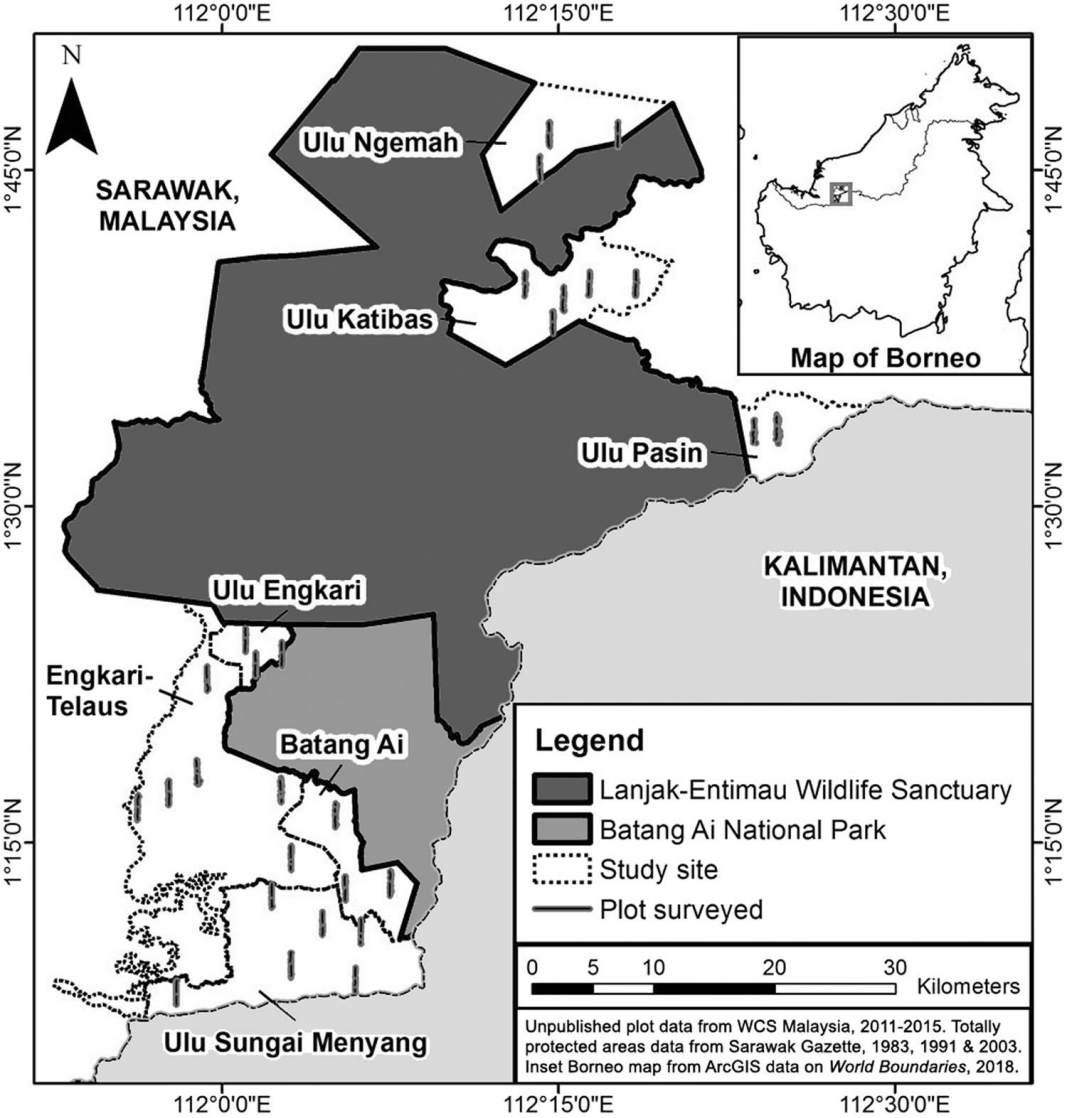
Map showing the study sites located adjacent to the two main protected areas (Lanjak-Entimau Wildlife Sanctuary and Batang Ai National Park), and the locations of plots surveyed throughout the survey duration from 2011 to 2015. This map was created using the software ArcGIS 10.2.1 (www.esri.com) by SN and JP.

The surveys reported in this paper were conducted at the greater Batang Ai-Lanjak-Entimau (BALE) landscape in the Malaysian state of Sarawak. The BALE landscape consists of two contiguous protected areas, namely the Batang Ai National Park (BANP) and the Lanjak-Entimau Wildlife Sanctuary (LEWS). The ‘greater’ BALE landscape refers to the seven sites surrounding the two protected areas that consists of five proposed extension areas (at the time of survey)^39^ and two non-protected landscapes (Fig. 2). The seven study sites surveyed in chronological order were Batang Ai, Ulu Engkari, Ulu Ngemah, Ulu Katibas, Ulu Pasin, Ulu Sungai Menyang, and Engkari-Telaus. The area sizes of the sites ranged from 22.48 km^2^ to 247.80 km^2^ and the total area combined was 680.21 km^2^ (Table 2). For further information on the study sites, refer to the Supplementary Appendix S2.

**Table 2:** The study sites and their area sizes.

Study site	Acronym	Protection status	Size (km^2^)
1. Batang Ai	BA	Proposed Southern extension of BANP	58.28
2. Ulu Engkari	UE	Proposed Northern extension of BANP	22.48
3. Ulu Ngemah	UN	Proposed extension of LEWS	69.40
4. Ulu Katibas	UK	Extension of LEWS (proposed at the commencement of surveys, became part of the sanctuary in 2013)	96.41
5. Ulu Pasin	UP	Extension of LEWS (proposed at the commencement of surveys, became part of the sanctuary in 2013)	45.84
6. Ulu Sungai Menyang	USM	Non-protected landscape	140.00
7. Engkari-Telaus	ET	Non-protected Community Conservation Landscape	247.80
		**Total area size=**	**680**.**21**

The protection status of each study site (proposed, gazetted as extension, or non-protected) is also indicated. The acronym BANP refers to Batang Ai National Park, and LEWS refers to Lanjak-Entimau Wildlife Sanctuary.

### Sampling scheme

In total, there were 42 plots placed at random at the study sites (Supplementary Table S1). The purpose of the random placement was to have a better representation of the heterogeneous nature of the greater BALE landscape to include valleys, streams and ridges. Each plot consists of four strips 36 m in width and 1 km in length arranged in a north-south direction (Supplementary Fig. S7). The survey extended 18 m from each side of the centre line. The use of 18 m half-width for the plot was derived from previous surveys using Distance sampling at the Batang Ai-Lanjak-Entimau (BALE) landscape between 2003 and 2007 whereby perpendicular distances (PPD) were truncated at 18 m.

We surveyed 29 of the plots and had the remaining 13 as reserves (Supplementary Figs S4 and S5). For the first five study sites, surveys were conducted at no less than three plots with signs of orang-utan nest. If one of the plots had no sign of old or new nest, the reserved plot would be surveyed. But we soon discovered three plots per study site were insufficient and opted to increase the number of plots to six per study site for the latter two study sites. Fieldwork commenced from January 2011 to September 2012, and subsequently continued from October 2013 to May 2015.

The Marked Nest Count (MNC) method^16,19^ is a survey technique used in estimating great ape population based on counting and marking new nests built within specific plot (known area size) during a specific time interval (known number of days). New nest is described as with green leaves, and old nest as without green leaf. We used four different nest decay classes adapted from van Schaik *et al*. to refer to nest classification (Supplementary Table S5). Old nests were observed at each plot and were later used in the Bayesian analysis to determine the probability of whether orang-utans used the plot previously or not.

Nest construction rate ($\hat{D}$) is one of the key features in the MNC. It refers to the density of new nests built between the first and the last survey at plots with known area sizes (nests km^−2^ day^−1^). To estimate $\hat{D}$, all nests were observed and new nests were marked on the first visit (*x*). This was to ensure that the latter were not included with the new ones built after the first survey (*y*). It was assumed that any new nests built after we visited the plot were still visible and were recorded as new 21 days later. The interval between repeat surveys was adequately short to ensure that no new nests constructed between the survey periods can disappear before the next survey^16^.

There were three main assumptions used in the survey methodology:

1. All orang-utan nests present at the plot were recorded on the first survey.
2. All new nests constructed in the plot after the first surveys were recorded on subsequent surveys.
3. Plots with no orang-utan nests (old/new) at the first survey were assumed never to be used or at least were not used in the next 42 days (the survey duration) by orang-utan, and nest construction rate was zero.

Additional information on Plot layout, steps for the First survey, followed by Second and Third surveys are found in Supplementary Appendix S3.

### Bayesian framework for the *N*-mixture models

Kruschke^29^ defines Bayesian analysis as the process of reallocating prior credibility consistent with the new data observed. Possibilities that were consistent with the data gain more credibility; possibilities that were not, lose credibility. The Bayesian framework is the structure where the reallocation takes place. All the possibilities were spread out as a probability distribution; thus, the total area under the histogram is equal to 1. The most credible range of possibilities which covers 95% of the posterior distribution is the highest density interval (HDI).

‘*N*’ refers to abundance. The term ‘mixture’ in our models is derived from the Binomial/Poisson mixture that we used to estimate abundance of new nests from signs of plot use by orang-utans based on the probability of detection by observers^31,33^. We did not use any covariates and opted to use an intercept-only model. We summarize the Bayesian analysis for the *N*-mixture models of this study into three steps (Supplementary Appendix S4 and Supplementary Fig. S8).

### Step 1: Describe the latent and observation processes

#### Latent process

Suitability model: Sign of plot use by orang-utans (*z*) is indicated by detection of old or new nest on the first surveys. An indicator variable *z*_*i*_ = 1 was used if signs of old orang-utan nest at plot *i* (*i* = 1, 2, 3, …, *N*) was available for detection. We had *z* = 0 for plots without signs of use (this is the zero-inflation part). The relationship for the model depends on a Bernoulli process (*z*_*i*_, detected or not detected) and varies among plots based on the probability of old nest at a site, *ψ*. The suitability model given plot use by orang-utans is shown in Eq. (1):

1 $$\,{z}_{i} \sim {\rm{Bernoulli}}\,({\psi }_{i})$$

As a cost effective measure, plots with *z* = 0 on the first survey were not revisited.

Abundance of new nests model: The purpose of this model is to find out how many new nests were built between the first and the last visit. The zero-inflated Poisson here captures the latent process of modelling expected new nests abundance (*n*_*i*_) given spatial variation and the suitability model (*z*_*i*_). This model is written in Eq. (2):

2 $$\,{n}_{i} \sim {\rm{Poisson}}\,({\lambda }_{i}\times {z}_{i}\times {a}_{i}\times {t}_{i}),\,{\rm{for}}\,{z}_{i}=1$$

Here, *λ* is the expected estimate of nest construction rate (nests km^−2^ day^−1^). We included plot size *a* (km^2^), and the time between the first and last survey *t* (days) for plots *i* to get number of nests without the rate units. The *λ* here refers to the expected nest construction rate if the whole study site was used by orang-utans (Supplementary Table S3 and Fig. S1). This rate is different from the estimated $\hat{D}$ which accounts for plots used and not used by orang-utans.

#### Observation process

Detection model: This model is based on a Binomial argument that assumes detection probability (*q*) is independent and identical for all expected abundance of new nests (*n*_*i*_) at plots *i*. This relationship is modelled in Eq. (3):

3 $${y}_{i} \sim {\rm{Binomial}}\,(q,{n}_{i})$$

where *q* is the probability of detecting new nest by two teams on the first survey. Only the total number of new nests recorded on the second and third surveys (*y*) is used in this model. The observation *y* will always be lower than the latent *n* due to imperfect detection. The number of new nests recorded on the first survey (*x*) was used to estimate imperfect detection *q*, as part of assessing Assumption #1. The relationship between *x* and *q* is further shown in Supplementary Table S4.

### Step 2: Generate estimates of nest construction rate, orang-utan density and population

#### Estimate of nest construction rate

We weighted the expected estimate of nest construction rate ($\hat{\lambda }$) with the probability of old nest at a site ($\hat{\psi }$) so as not to lose information about habitat use by orang-utans. We show the estimated nest construction rate ($\hat{D}$) for the whole study site, used and not used by orang-utans in Eq. (4):

4 $${\hat{D}}_{M}={\hat{\lambda }}_{M}\times {\hat{\psi }}_{M}$$

where *M* is the study site in chronological order: Batang Ai, Ulu Engkari, … Engkari-Telaus (*M* = 1, 2, … 7).

#### Estimates of orang-utan density and population

After generating the estimated nest construction rate, we determine the estimated orang-utan density, $\hat{d}$ (orang-utan km^−2^) at the study site using Eq. (5):

5 $${\hat{d}}_{M}=\frac{{\hat{D}}_{M}}{\hat{p}\times \hat{r}}$$

where, $\hat{p}$ = estimated proportion of nest builders in the population.

$\hat{r}$ = estimated daily rate at which nest-builders build nests (nests orang-utan^−1^ day^−1^).

$\hat{p}\times \hat{r}$ = number of nests built per orang-utan per day.

Orang-utan follows to determine $\hat{p}\times \hat{r}$ for the BALE landscape was unsuccessful in past attempts which were conducted from September to November 2006^38^. Instead, we used the results by Ancrenaz *et al*.^22^ for the values of $\hat{p}$ ≈ 0.85 (given *n* = 92 individuals) and $\hat{r}$ ≈ 1.00 (given 602 dawn-to-dusk follows) obtained at Kinabatangan, Sabah.

We then determine the estimated orang-utan population at each study site throughout the duration of the survey ($\hat{\mu }$) using Eq. (6):

6 $$\begin{array}{c}{\hat{\mu }}_{M}=\,{\hat{d}}_{M}\,\times {\rm{A}}{\rm{r}}{\rm{e}}{\rm{a}}\,{\rm{s}}{\rm{i}}{\rm{z}}{\rm{e}}\,{\rm{o}}{\rm{f}}\,{\rm{s}}{\rm{t}}{\rm{u}}{\rm{d}}{\rm{y}}\,{{\rm{s}}{\rm{i}}{\rm{t}}{\rm{e}}}_{M}\,({{\rm{k}}{\rm{m}}}^{2})\end{array}$$

### Step 3: Assess strength or reliability of the estimates

#### Identifiability test

This test by Gimenez *et al*.^30^ is a simple guideline to check the identifiability of any parameter of interest (*θ*) by declaring it weak for overlap of >35% between the marginal prior and its marginal posterior distributions. The percentage overlap (*τ*_*θ*_) between the two distributions for data *Y* is shown in Eq. (7):

7 $${\tau }_{\theta }=\int {\rm{\min }}(p(\theta ),\,\pi (\theta |Y){\rm{d}}\theta )$$

where *p(θ)* is the marginal prior distribution, and *π(θ|Y)* is the marginal posterior distribution. The estimates for the parameter *θ* is said to be weak and misleading when *π(θ|Y)* ≈ *p(θ)*.

### Implementation in programme R

We used the Markov chain Monte Carlo (MCMC)^40^ to perform the Bayesian analysis for our *N*-mixture models and to estimate uncertainty for better interpretation of the results. We ran the estimation in Just another Gibbs sampler or JAGS^40^ using the R2jags interface^41^ in programme R (version 3.4.3)^42^. We used the coda package developed by Plummer *et al*.^43^ and R2jags package version 0.5–7 developed by Su & Yajima^41^ to run the models.

We used uninformative priors: a uniform [0, 1] prior for $\hat{\psi }$, and a uniform [0, 4] prior for $\hat{\lambda }$. Prior sensitivity analysis showed that the latter was uninformative. The analysis converged quickly, and 40,000 iterations were used, of which 5,000 were discarded as burn-in. The mean and 95% highest density interval (HDI) of the MCMC samples were used to summarise the posterior probabilities.

In order to assess the strength and reliability of the generated estimates, the posterior and prior distributions were made to overlap using the function postPriorOverlap within the wiqid package version 0.1.3 developed by Meredith^44^. The complete R scripts for these analyses are shown in Supplementary Appendix S5.

### Use Of experimental animals and human subjects

No experiments on live vertebrates or human subjects were performed for this paper. All survey protocols in the Methods section were approved by the Wildlife Conservation Society in accordance with the approved guidelines.

## Electronic supplementary material

Supplementary Materials
