# Supplementary material for: Population estimates of Bornean orang-utans using Bayesian analysis at the greater Batang Ai-Lanjak-Entimau landscape in Sarawak, Malaysia
Source: Sci Rep. 2018 Oct 23;8:15672. doi: 10.1038/s41598-018-33872-3 (PMC6199283; doi:10.1038/s41598-018-33872-3)
Supplement: Supplementary file 1 — Supplementary Materials [file 41598_2018_33872_MOESM1_ESM.pdf]

COVER PAGE

TITLE: Population estimates of Bornean orang-utans using Bayesian analysis at the greater Batang Ai-Lanjak-Entimau landscape in Sarawak, Malaysia

Primary and Corresponding author:
Joshua Pandong[1,2],
[1]Wildlife Conservation Society (WCS)-Malaysia Programme, No. 7 Jalan Ridgeway, 93200 Kuching, Sarawak, Malaysia.
Present address: [2]School of Biological Sciences, The University of Adelaide, South Australia 5005, Australia
E-mail: jpandong@wcs.org
Tel (O): +6082-239795, +6013-8018713
Fax (O): +6082-239578

Additional authors:
Melvin Gumal[1],
[1]Wildlife Conservation Society (WCS)-Malaysia Programme, No. 7 Jalan Ridgeway, 93250 Kuching, Sarawak, Malaysia. E-mail: mgumal@wcs.org

Lukmann Alen[1,3],
[1]Wildlife Conservation Society (WCS)-Malaysia Programme, No. 7 Jalan Ridgeway, 93250 Kuching, Sarawak, Malaysia during the field surveys. Currently with
[3]WWF Malaysia, Bangunan Binamas 7th Floor, Jalan Padungan, Kuching, Sarawak, Malaysia. E-mail: LAlen@wwf.panda.org

Ailyn Sidu[1,3],
[1]Wildlife Conservation Society (WCS)-Malaysia Programme, No. 7 Jalan Ridgeway, 93250 Kuching, Sarawak, Malaysia during the field surveys. Currently with
[3]WWF Malaysia, Bangunan Binamas 7th Floor, Jalan Padungan, Kuching, Sarawak, Malaysia. E-mail: asidu@wwf.panda.org

Sylvia Ng[1],
[1]Wildlife Conservation Society (WCS)-Malaysia Programme, No. 7 Jalan Ridgeway, 93250 Kuching, Sarawak, Malaysia. E-mail: sng@wcs.org

Lian Pin Koh[2,4],
[2]School of Biological Sciences, The University of Adelaide, South Australia 5005, Australia.
[4]Conservation International, 3131 East Madison Street, Suite 201, Seattle, WA 98112, USA.
E-mail: lianpinkoh@gmail.com

Supplementary materials:

TITLE: Population estimates of Bornean orang-utans using Bayesian analysis at the greater Batang Ai-Lanjak-Entimau landscape in Sarawak, Malaysia

Supplementary Appendix S1: Additional data and results.

Supplementary Table S1. Overview of plots surveyed and reserved for the study sites. The site where the survey teams had to use the reserves is highlighted in gray.

**Study site**	**No. of plots surveyed**	**No. of plots reserved**	**Surveyed plot ID**	**Reserved plot ID**
Southern (Batang Ai)	3	5	A,B,C	D,E,F,G,H
Northern (Ulu Engkari)	3	2	I,J,K	L,M
Ulu Ngemah	3	2	N,O,P	Q,R
Ulu Katibas	5	0	S,T,U,V,W	-*
Ulu Pasin	3	2	X,Y,Z	AA,AB
Ulu Sungai Menyang	6	1	A1,B1,C1,D1,E1,F1	G1**
Engkari-Telaus	6	1	A2,B2,C2,D2,E2,F2	G2**
**Total:**	**29**	**13**		

Notes:
*As two of the reserves were used (Plots V and W), they are not indicated under the column 'Reserved plot ID'.
**Plots G1 and G2 were not surveyed as permission was not granted by local communities.

Supplementary Table S2. Details for three repeat surveys conducted at the study sites at the greater BALE landscape. Coordinate system: Timbalai 1948 (RSO Borneo Meters).

Plot	Area	Location	GPS coordinates for the centre of each plot		Dates for repeat surveys			Num. of days, *t*	New nests recorded on the first survey, *x*	Total new nests on second and third surveys, *y*	Sign of orang-utan nest (old/ new), *z*	Plot size, *a* (km^2)
			Easting (m)	Northing (m)	First	Second	Third					
A	BA	Nanga Senyumboh	271059	135000	18 & 19 Mar. 2011	8 Apr. 2011	29 Apr. 2011	40	2	7	1	0.1563
B	BA	Mawang-Wong Tibang	267334	134560	22 & 23 Mar. 2011	11 Apr. 2011	3 May 2011	40	10	9	1	0.1552
C	BA	Semuban	266527	140567	26 & 27 Mar. 2011	14 Apr. 2011	6 May 2011	40	2	6	1	0.1474
I	UE	Engkramoh Ulu	262079	153833	7 & 8 Jul. 2011	30 Jul. 2011	20 Aug. 2011	42	0	6	1	0.1464
J	UE	Engkramoh Ili	259925	152962	12 & 13 Jul. 2011	1 Aug. 2011	22 Aug. 2011	39	2	9	1	0.1446
K	UE	Segrak	259080	155009	18 & 19 Jul. 2011	5 Aug. 2011	25 Aug. 2011	37	0	11	1	0.1384
N	UN	Empurau	289818	196553	12 & 13 Nov. 2011	-	-	2	NA	NA	0	0.1448
O	UN	Pang	284175	196558	10 Dec. 2011	-	-	1	NA	NA	0	0.1483
P	UN	Semujan	283378	193744	16 Dec. 2011	-	-	1	NA	NA	0	0.1442
S	UK	Likau	291331	184295	17 & 18 Feb. 2012	-	-	2	NA	NA	0	0.1544
T	UK	Katibas	285377	183037	21 & 22 Feb. 2012	-	-	2	NA	NA	0	0.1643
U	UK	Datai	287468	184320	25 & 27 Feb. 2012	19 Mar. 2012	7 Apr. 2012	42	1	1	1	0.1434
V	UK	Nyungan	284482	181015	13 & 15 Mar. 2012	4 Apr. 2012	25 Apr. 2012	40	4	1	1	0.1465
W	UK	Begua	282162	184228	17 & 18 Mar. 2012	6 Apr. 2012	-	19	0	0	1	0.1269
X	UP	Bloh Karoh	301123	172139	19 & 20 Jul. 2012	10 Aug. 2012	1 Sep. 2012	42	1	7	1	0.1440
Y	UP	Selemas	303123	172139	21 & 22 Jul. 2012	13 Aug. 2012	3 Sep. 2012	41	4	5	1	0.1496
Z	UP	Selemas	302982	172487	25 & 26 Jul. 2012	16 Aug. 2012	5 Sep. 2012	40	0	4	1	0.1566
A1	USM	Genting Badak	268196	127059	22 & 23 Mar. 2014	13 Apr. 2014	4 May 2014	43	0	2	1	0.1248
B1	USM	Kasai	268628	131014	29 & 30 Mar. 2014	15 Apr. 2014	6 May 2014	38	3	6	1	0.1371
C1	USM	Jambu	265456	131678	2 & 3 Apr. 2014	16 Apr. 2014	8 May 2014	36	1	6	1	0.1387
D1	USM	Ulu Jirak	262849	128260	13 & 14 Mar. 2015	8 Apr. 2015	1 May 2015	49	4	4	1	0.1507
E1	USM	Sumpa	261248	133965	6 & 7 Apr. 2015	27 & 28 Apr. 2015	22 May 2015	46	2	4	1	0.1445
F1	USM	Kedang Katik	253358	126012	20 & 21 Oct. 2013	11 Nov. 2013	1 Dec 2013	42	0	0	1	0.1441

Plot	Area	Location	GPS coordinates for the centre of each plot		Dates for repeat surveys			Num. of days, *t*	New nests recorded on the first survey, *x*	Total new nests on second and third surveys, *y*	Sign of orang-utan nest (old/ new), *z*	Plot size, *a* (km^2)
			Easting (m)	Northing (m)	First	Second	Third					
A2	ET	Nanga Suga	255082	144128	22 & 23 Sep. 2014	15 Oct. 2014	-	22	0	0	1	0.1575
B2	ET	Engkramoh	255898	151829	13 & 14 Apr. 2015	14 Apr. 2015	25 May 2015	42	1	3	1	0.1409
C2	ET	Senibong	262841	137065	13 & 14 Mar. 2015	11 Apr. 2015	3 May 2015	51	2	2	1	0.1475
D2	ET	Tisau Ulu	252726	142431	22 & 23 Oct. 2014	-	-	1	NA	NA	0	0.1595
E2	ET	Sungai Tutong	250149	141145	28 & 29 Oct. 2014	-	-	1	NA	NA	0	0.1519
F2	ET	Ukap	262022	142650	19 & 20 Mar. 2015	13 Apr. 2015	5 May 2015	46	1	0	1	0.1569
				**Total=**	**29**	**22**	**20**	**-**	**40**	**93**	**22**	**4.2654**

Note: There were no repeat surveys conducted on the second and third occasions for Plots N, O, P, S, T, D2 and E2 (shown as '-') as there was no sign of orang-utan nest (old/new) in the plots. In results for *y*, 'NA' refers to no sign of orang-utan nests (old/new) at the plot, and '0' refers to old orang-utan nests detected at the plot but no new nests recorded.

Supplementary Table S3. Estimates of nest construction rate, $\hat{D}$ (nests km^{-2} day^{-1}), expected nest construction rate if whole study site is used by orang-utans ($\hat{\lambda}$), and probability of old nest at a site ($\hat{\psi}$) with 95% highest density interval (HDI) for the study sites at the greater BALE landscape.

Study site	Area	Estimated nest construction rate (given plot use by orang-utans)			Expected estimate of nest construction rate			Estimated probability of old nest at a site		
		$\hat{D}$	Lower HDI	Upper HDI	$\hat{\lambda}$	Lower HDI	Upper HDI	$\hat{\psi}$	Lower HDI	Upper HDI
Southern (Batang Ai)	BA	1.1942	0.4624	1.9198	1.4905	0.8585	2.1889	0.8011	0.4765	1.0000
Northern (Ulu Engkari)	UE	1.5206	0.6326	2.4363	1.9008	1.1423	2.7501	0.8003	0.4743	1.0000
Ulu Ngemah	UN	0.3979	0.0000	1.3342	1.9988	0.0148	3.8142	0.1995	0.0000	0.5269
Ulu Katibas	UK	0.1461	0.0068	0.3412	0.2559	0.0273	0.5552	0.5715	0.2381	0.8986
Ulu Pasin	UP	0.8648	0.3130	1.4402	1.0814	0.5653	1.6554	0.8000	0.4699	1.0000
Ulu Sungai Menyang	USM	0.7008	0.3444	1.0671	0.8009	0.4641	1.1769	0.8750	0.6497	1.0000
Engkari-Telaus	ET	0.1932	0.0369	0.3885	0.3082	0.0908	0.5735	0.6265	0.3250	0.9267

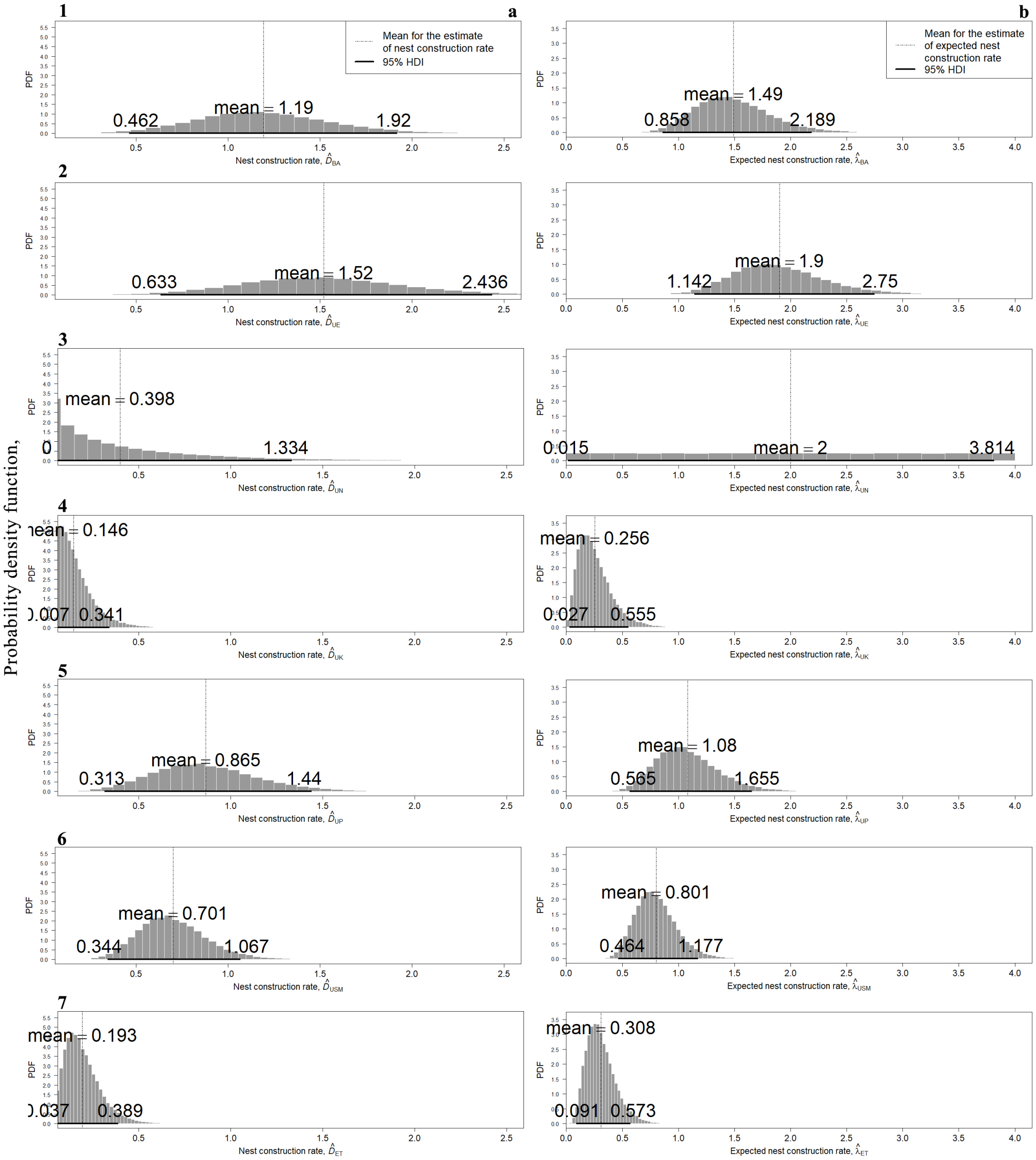

Supplementary Figure S1. The columns correspond to the probability density function of: a) estimated nest construction rate ($\hat{D}$), and b) expected estimate of nest construction rate ($\hat{\lambda}$) with 95% HDI at the study sites. The row sequence corresponds to the study sites: 1) Batang Ai, 2) Ulu Engkari, 3) Ulu Ngemah, 4) Ulu Katibas, 5) Ulu Pasin, 6) Ulu Sungai Menyang, and 7) Engkari-Telaus.

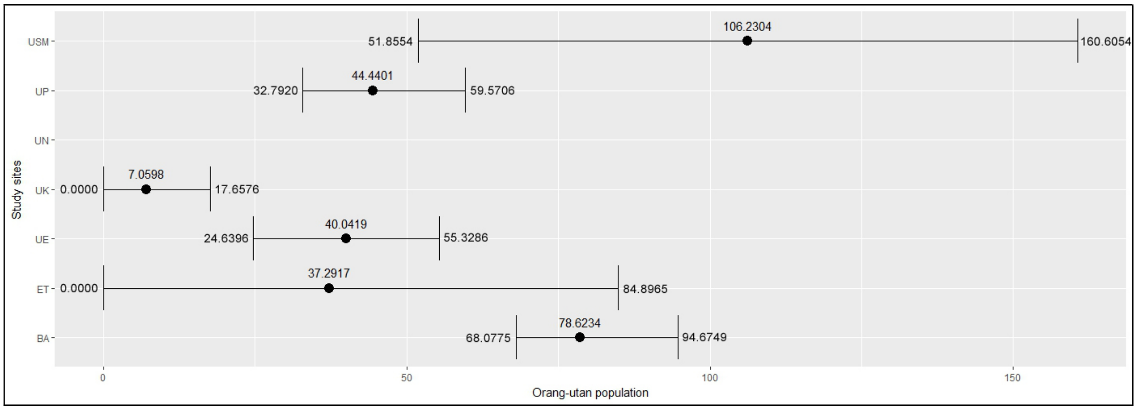

Supplementary Figure S2. Orang-utan population estimates based on a non-Bayesian approach with 95% confidence interval (CI). We conducted bootstrapping analysis with 100,000 random sampling with replacement of the original datasets shown in Supplementary Table S2. The methodology for the analysis was adapted from Ancrenaz et al [1]. The confidence interval here is shown as a line with two end points as the results do not correspond to a probability distribution, but based on a sampling distribution with area under the curve that do not integrate to 1.

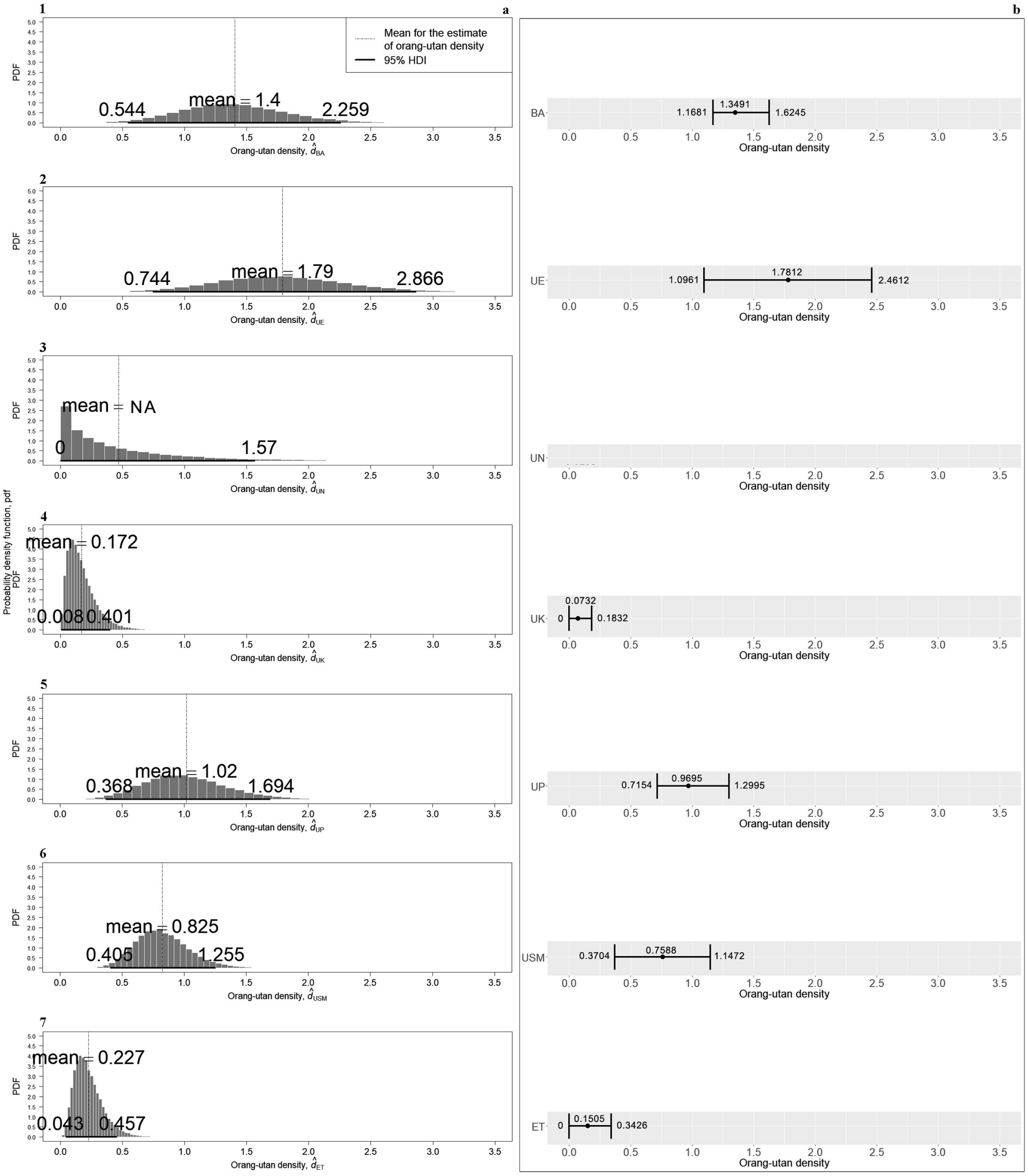

Supplementary Figure S3. Estimated orang-utan density based on the: a) Bayesian method with 95% HDI, and b) non-Bayesian approach with 95% CI. The row sequence corresponds to the study sites: 1) Batang Ai, 2) Ulu Engkari, 3) Ulu Ngemah, 4) Ulu Katibas, 5) Ulu Pasin, 6) Ulu Sungai Menyang, and 7) Engkari-Telaus. The lower limit of orang-utan density ranges at sites with low counts of new nest should be > 0. However, the bootstrap analysis (non-Bayesian approach) could not compute low counts of new nests at sites UK and ET, resulting in 0 included within the 95% CI. This is incorrect as there were new nests recorded, meaning at least 1 orang-utan was present at each site at the time of survey.

Supplementary Appendix S2: Background of study sites, and location of surveyed plots and new orang-utan nests at the study sites.

Background of study sites

Malaysia comprises of three regions: Peninsular Malaysia, Sabah and Sarawak. It is in the latter two regions that orang-utans are found. In Sarawak, orang-utans are only found in two locations: the Batang-Ai-Lanjak-Entimau (BALE) landscape where the main populations are found and the Gunung Lesong-Ulu Sebuyau-Sedilu landscape where the remnant populations are still sighted. Orang-utans are not found in other areas including Maludam National Park, even after surveys in 1985, 1987, 1988, 1990 and 1994 [2,3] and the exhaustive 18-month research in the area between 2002 and 2004 [4,5]. There were reports of orang-utan sightings in the past 10 years at Bungo Range National Park, Sabal Forest Reserve and the proposed Klingkang Range National Park. Researchers from the WCS Malaysia conducted rapid assessments to document orang-utan signs at these areas in 2016 and 2017 but no signs detected to date.

The BALE landscape consists of two contiguous protected areas, namely the Batang Ai National Park (BANP) and the Lanjak-Entimau Wildlife Sanctuary (LEWS). The greater BALE landscape then consists of seven areas: five proposed extension areas (at the time of survey) [6] and two non-protected landscapes. After completing surveys at Ulu Katibas and Ulu Pasin, both areas were successfully gazetted as extensions to Lanjak-Entimau Wildlife Sanctuary in May 2013.

Prior to the surveys, anecdotal information shows that there were orang-utan sightings in Ulu Ngemah, Ulu Katibas and Ulu Pasin [6]. These areas were then considered for potential extensions to reduce commercial exploitation and encroachment into lands with community rights [6]. Although the areas consist of native forests, they could have been alienated for development, be it for logging, or large-scale agriculture, as they were not part of LEWS. The three extension areas of LEWS are important for conservation as the current boundary is a cut line and does not follow natural ridgelines or the water catchment.

Similarly, in Batang Ai National Park, orang-utans were frequently sighted in the two sites outside the park: the proposed Southern extension (Batang Ai) and the Northern extension (Ulu Engkari) [7]. The proposed additions to Batang Ai National Park or proposed conservation areas were significant for conservation based on prior evidence of orang-utan presence in these places [7]. Rapid assessments carried out at Batang Ai in 2003 showed that the highest concentration of orang-utan nest surveyed was located there [8]. Repeat surveys in Lanjak-Entimau Wildlife Sanctuary along the periphery of Ulu Engkari also revealed significant number of orang-utan nests in the area [8].

Since 2012, researchers jointly conducted rapid assessments of new orang-utan nests at the Ulu Sungai Menyang landscape (2012 and 2013) and the Engkari-Telaus Community Conservation Landscape (2014) under the Heart of Borneo (HoB) Initiative [9]. The surveys conducted in the two non-protected landscapes were the direct result of threats from large-scale land use conversion. Licenses to log both landscapes were not renewed by the Director of Forests with directives from the State's Second Minister of Resource Planning and Environment (the Chief Minister of Sarawak has since renamed this ministry as 'Ministry of Urban Development and Natural Resources' in 2017).

The combined total area of the seven study sites is 680.21 km^2.

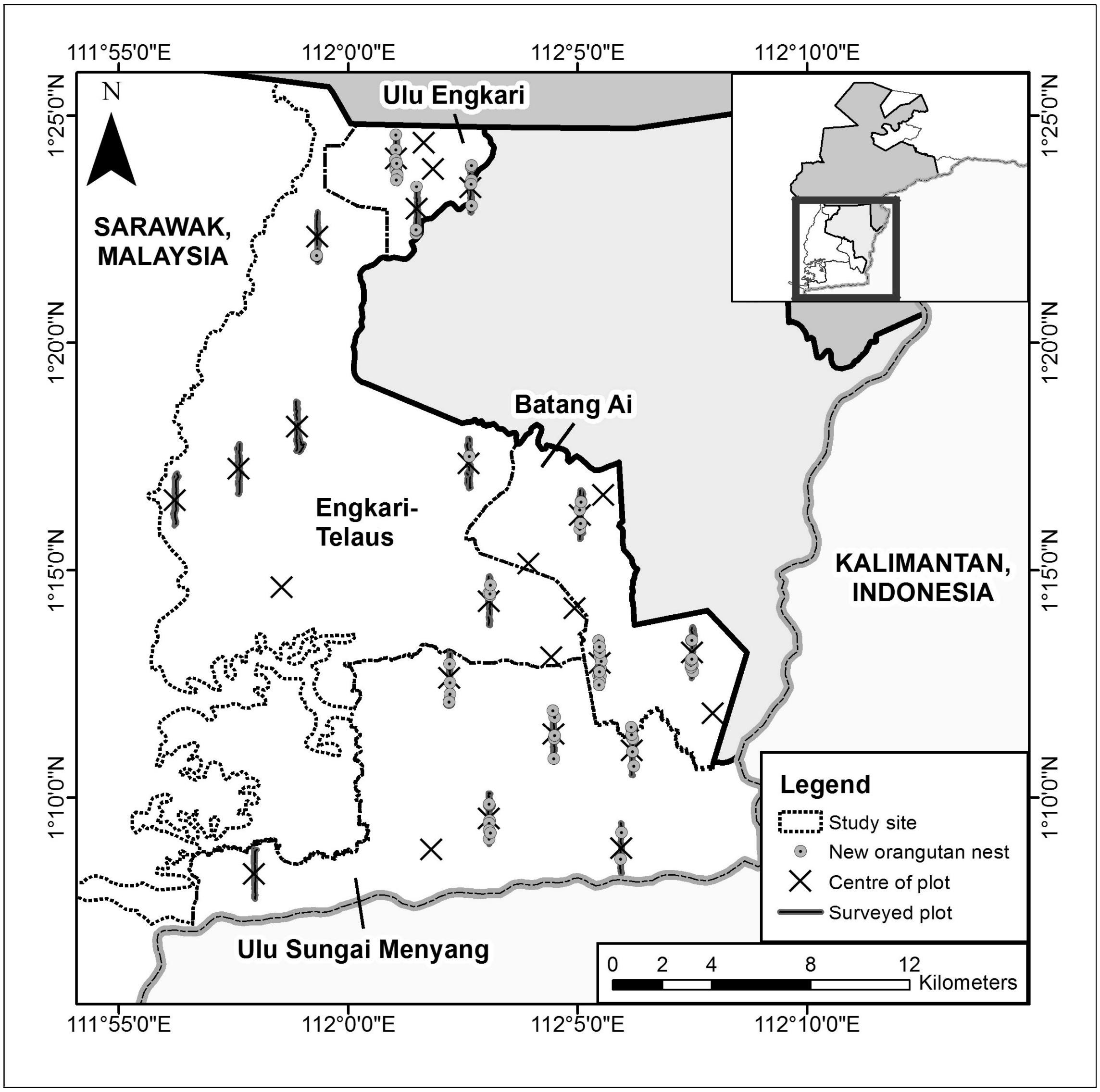

Supplementary Figure S4. Location of surveyed plots and new orang-utan nests at Ulu Engkari, Batang Ai, Engkari-Telaus and Ulu Sungai Menyang. The study sites are contiguous with the core habitats of Batang Ai National Park (light grey) and Lanjak-Entimau Wildlife Sanctuary (dark grey). Coordinate system: GCS WGS 1984 (WGS 1984). This map was created using the software ArcGIS 10.2.1 (www.esri.com) by SN and JP.

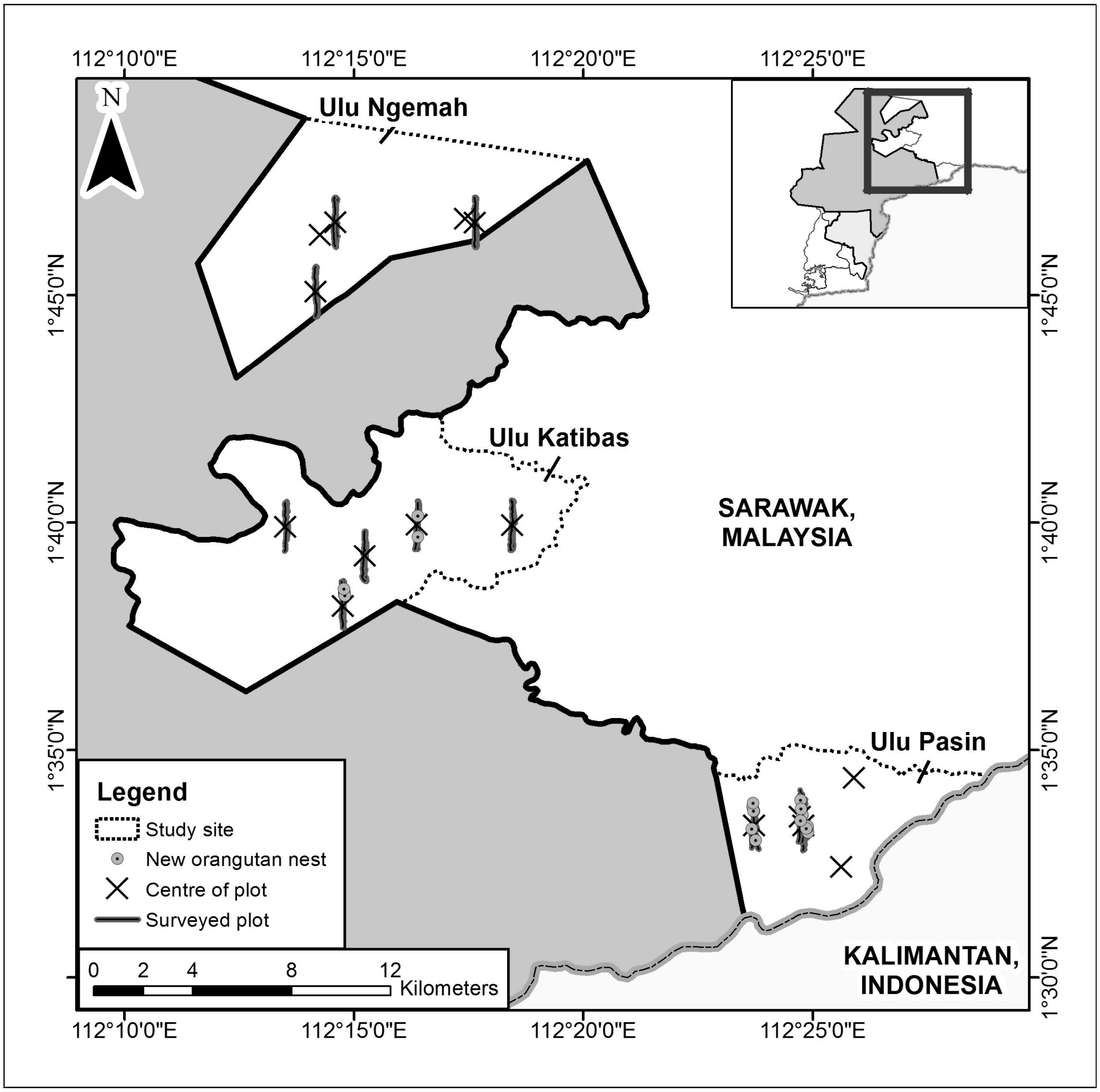

Supplementary Figure S5. Location of surveyed plots and new orang-utan nests at Ulu Ngemah, Ulu Katibas and Ulu Pasin. The study sites are contiguous with the core habitat Lanjak-Entimau Wildlife Sanctuary (dark grey). Coordinate system: GCS WGS 1984 (WGS 1984). This map was created using the software ArcGIS 10.2.1 (www.esri.com) by SN and JP.

Supplementary Appendix S3: Additional method description.

a) Plot layout

We selected the centre for each plot (both easting and northing) at random using `=RANDBETWEEN(bottom, up)` function in MS Excel. The values for `(bottom, up)` were the edges of a rectangular connecting the boundary edges of the extension area. If the randomly selected centre of plot fell outside the study area, it was rejected. Only plot centres inside the study site and at least 1 km away from nearby protected areas or boundary of the study site were selected.

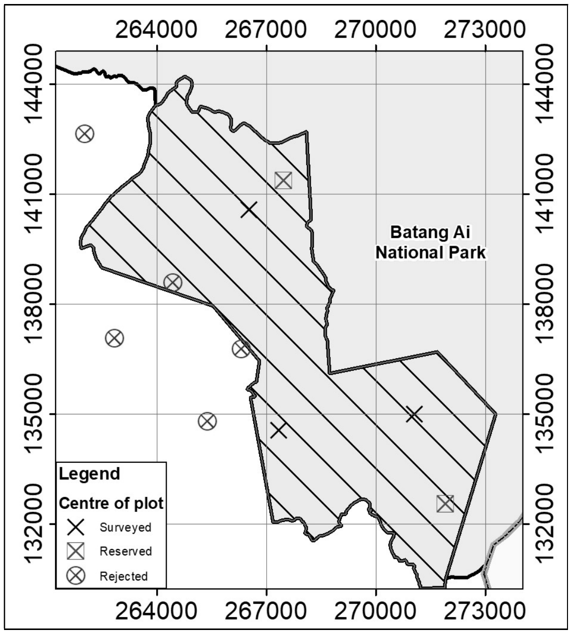

Supplementary Figure S6. The proposed Southern extension of Batang Ai National Park (hatching). Coordinate system: Timbalai 1948 (RSO Borneo Meters). This map was created using the software ArcGIS 10.2.1 (www.esri.com) by SN and JP.

Each plot consists of four strips 36 m in width and 1 km in length arranged in a north-south direction. A trail or *rentis* was marked along the centre line of each strip before the surveys commenced. The survey extended 18 m from each side of the *rentis*. The use of 18 m half-width for the plot was derived from previous surveys using Distance sampling at the Batang Ai-Lanjak-Entimau (BALE) landscape between 2003 and 2007 whereby perpendicular distances (PPD) were truncated at 18 m.

We identified four strips by their positions: Northwest, Northeast, Southwest and Southeast respectively. The rationale of having four strips was to increase survey effort in one plot and save travelling time between plots, instead of having multiple two-strip plots situated far apart from each other.

The sampling scheme of this study design included the following:

i. All the plots were named alphabetically. The order of the alphabets operates as the sequence for travelling between plots (where possible);
ii. Surveys were conducted at no less than three plots with sign of orang-utan nest (old/new);
iii. If one of the plots had no sign of nests, the reserve plot would be surveyed. This was only limited to the two reserve plots;

iv. Surveys were stopped (no second and third surveys) if all the three original plots or six original plots had no sign of any orang-utan nest (old/new). The three original plots refer to Batang Ai (BA), Ulu Engkari (UE), Ulu Ngemah (UN), Ulu Katibas (UK) and Ulu Pasin (UP). But subsequently, we realized that a minimum of three plots for each study site were too few. Therefore, we opted to increase original plots to be surveyed to six plots for Ulu Sungai Menyang (USM) and Engkari-Telaus (ET). Surveys were also stopped if three plots (or six plots for USM and ET) with the two reserves (due to absence of orang-utan nests in one or two of the original plots) had no sign of orang-utan nest (old/new)*;

v. Given budgetary constraints, labour restrictions and limited time, the maximum numbers of plots that could be surveyed were up to five for BA, UE, UN, UK and UP, and up to seven plots for USM and ET.

*NOTE: The actual sampling scheme was as follows: only three plots were surveyed at BA, UE, UN and UP, five plots at UK, and six plots each for USM and ET. No orang-utan nest (new or old) was observed during the first survey at Plots S and T in UK (Supplementary Table S1). These two plots were then designated as not having used by orang-utans and not resurveyed. Meanwhile, new orang-utan nest was recorded at the third plot (U) on the first survey at UK. Therefore, two reserved plots (Plots V and W) were surveyed in replacement for Plots S and T. New orang-utan nests were seen in these reserved plots at UK. The plots at UN were discontinued altogether as no new or old orang-utan nests were recorded in all three plots (N, O and P).

b) First survey

We surveyed each strip thoroughly twice, with two separate teams walking in opposite directions. The manner for the search was: Team 1 surveyed Strip-NW first, and then re-surveyed by Team 2 from the opposite direction on the same day. Meanwhile, Team 2 surveyed Strip-NE first, and then by Team 1 from the opposite direction. The same applies for Strip-SW and Strip-SE by both teams (Supplementary Figure S7).

We recorded new orang-utan nests within the strips (18 m each side of the *rentis*) and clearly tagged the trees that had these nests. Wich & Boyko [10] noted that 'there was a sharp drop in nest detection after 10 m on either side of the line transect', and 'nests beyond 10 m were found less than half the time by every team'. However, it must also be noted here that Wich & Boyko's line transect method required the survey teams to stay on the *rentis*, while the plot count method for this project allowed the survey teams to fan out up to 18 m on either side. As such, the drop in nest detection after 10 m on either side of the one transect was not violated or relevant. Perpendicular distance (PPD) from the new nest to the centre line (*rentis*) was measured for verification. New orang-utan nests that were visible outside the strip were also recorded into the datasheet but not used for data analysis. The information was useful during subsequent surveys as a reference to avoid recording trees with new orang-utan nests that were outside the strip.

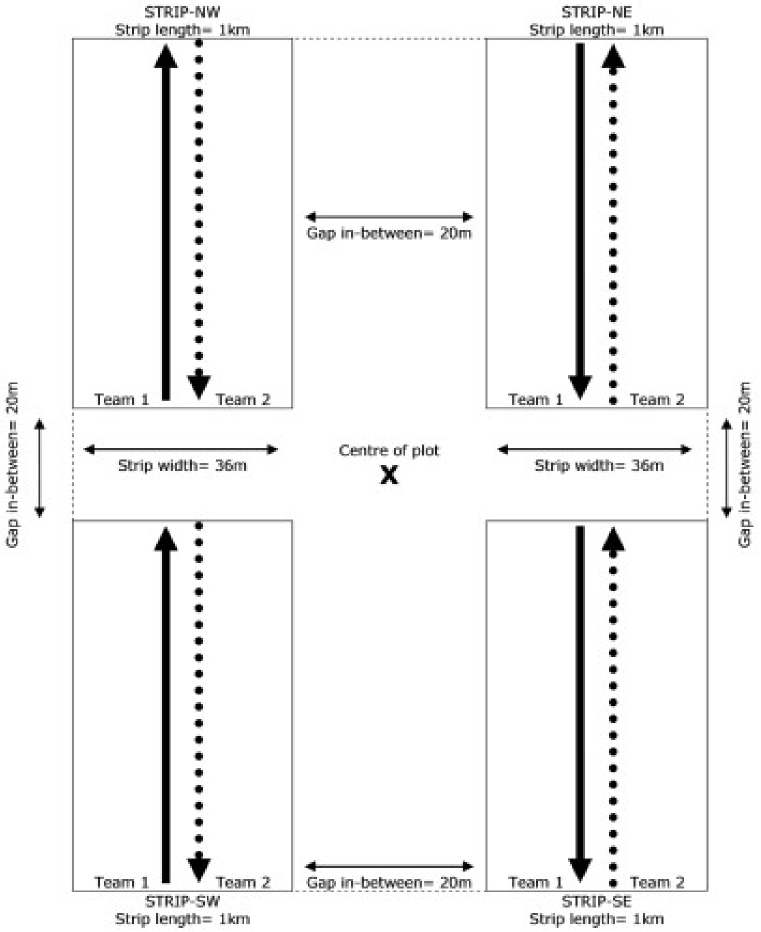

Supplementary Figure S7. Diagram of four strips in one plot.

The reasons for two teams on the first survey were: (a) to increase the probability of detecting all the orang-utan nests, and (b) to evaluate the assumption that all new nests were detected on a single search. New nests recorded on the first surveys were included in an analysis to assess *q*. These new nests were however not included in analyses of nest construction rate or for calculating the density of orang-utans in the plots as it was not known when these nests were constructed.

The two teams were not independent, as the first team tagged trees where they detected new nests. The second team could see the tagged trees and searched for, and recorded any additional new nests which the first team did not record.

Supplementary Table S4. Formula to evaluate assumption that all new nests were detected on a single search. Given: *q* is the probability of detecting new nest by two teams on the first survey, and; *x* is the number of new nests detected during the first surveys at each of the plots.

Description of first survey	Probability of detection	Estimated number of new nests detected during first surveys
i. New nest detected by the first team	q	$(q)\,x$
ii. New nest missed by the first team, but detected by the second team	$(1 - q)\,q$	$(1 - q)\,(q)\,x$
iii. New nest missed by the second team	$(1 - q)\,(1 - q)$	$(1 - q)\,(1 - q)\,x$

Orang-utan nests were described into two broad categories for this project: a new orang-utan nest has green leaves (at least one green leaf), whereas an old orang-utan nest is without green leaf. The two categories were further classified into four decay classes (Class A to Class D). During the surveys, only new nests (with green leaves), that is Class A and Class B nests, were recorded and used for data analysis.

Supplementary Table S5. Decay classes and categories for orang-utan nests adapted from van Schaik et al.[11].

Class	Nest category		Description
A	*With green leaves*	:	New; leaves are still green
B		:	New but decaying; leaves may still be attached and the nest is still firm and solid
C	*No green leaf*	:	Old; leaves are gone and holes are visible in the nests
D		:	Very old; twigs and branches are still present, but no longer in the original shape of the nest

c) Second and third surveys

We conducted the second and third surveys for each plot at intervals of approximately 21 days. Each strip was only surveyed once by a single team as q was already estimated. The team searched the whole area for new nests and there was no second team to repeat the survey in the opposite direction. The same process was repeated for the third survey. The lead researchers for all the teams were the same throughout the project.

Supplementary Appendix S4: The 3-step guide to analysing the *N*-mixture models using a Bayesian framework.

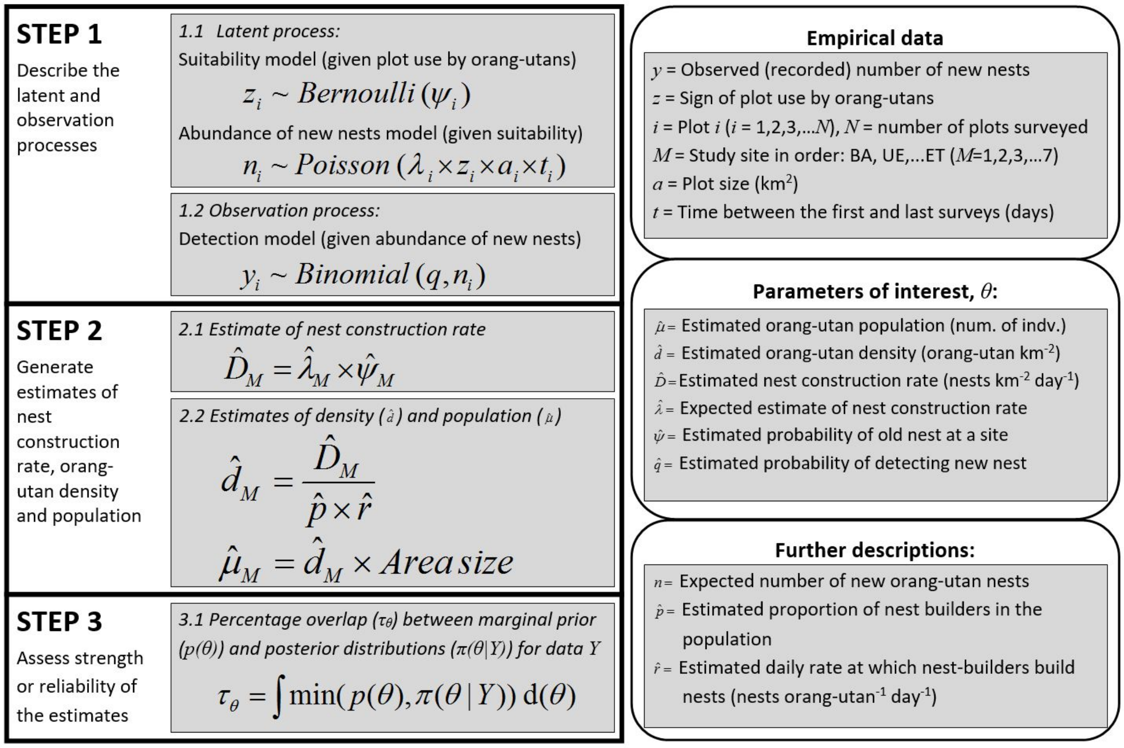

Supplementary Figure S8. Overview of the three-step guide to analysing the *N*-mixture models using a Bayesian framework [12] to generate estimates of nest construction rate, orang-utan density and population. The strength or reliability of the estimates was assessed using an identifiability test [13].

Supplementary Appendix S5. Data analysis (gray cells) and selected outputs (white cells) using JAGS in R.

Introduction

We used the Just Another Gibbs Sampler (JAGS) [14] in R [15] to perform the Markov chain Monte Carlo (MCMC) computation in the Bayesian analysis to generate the following:

1. Estimates of orang-utan population (mu.hat or $\hat{\mu}$), density (d.hat or $\hat{d}$) and probability of old nest at a site (psi or $\hat{\psi}$) with 95% highest density interval (HDI).
2. Histograms of the posterior outputs.

We surveyed seven study sites. The analysis sequence and results for this Appendix were in chronological order of the surveys, namely: 1. Batang Ai (BA); 2. Ulu Engkari (UE); 3. Ulu Ngemah (UN); 4. Ulu Katibas (UK); 5. Ulu Pasin (UP); 6. Ulu Sungai Menyang (USM); 7. Engkari-Telaus (ET).

Data analyses using the Bayesian framework

Objective #1: To generate estimates of orang-utan population (mu.hat or $\hat{\mu}$), density (d.hat or $\hat{d}$) and probability of old nest at a site (psi or $\hat{\psi}$) with 95% highest density interval (HDI).

1.1. <u>Load R packages and retrieve data</u>

Load two R packages for the analyses, namely: the `R2jags` package developed by Su & Yajima [16]; and the `wiqid` package developed by Meredith [17].

```
library(R2jags)
citation(package="R2jags")
library(wiqid)
citation(package="wiqid")
```

Retrieve the data shown in Supplementary Table S2 in .csv format. Area sizes in km^2 for the study sites are assigned in chronological order of the surveys (BA, UE, UN, UK, UP, USM, ET). The number of nest built per orang-utan per day is based on Ancrenaz et al's [1] as previous orang-utan follows were unsuccessful to determine $\hat{p} \times \hat{r}$ in the BALE landscape.

```
nests.Data.ALL <- file.choose()  # Retrieve data in .csv format
Area.ALL <- c(58.28, 22.48, 69.40, 96.41, 45.84, 140.00, 247.80)
p.hat_r.hat = 0.8500
```

1.2. <u>Run model</u>

Assign a `set.seed` to specify, save and restore the model. Priors used are broad uniform for $\hat{x}_0$, and `dbeta(5,1)` for q, that is to skew the distribution towards 1 in the probability density function. Details of the model are given in the Methods section of the paper and an overview of the 3-step guide to analysing the N-mixture models is shown in Supplementary Figure S8.

```
set.seed(123)
sink("model.txt")
cat("
model {
       # PRIORS:
       x0 ~ dunif(0, 100)
       x <- trunc(x0)
```

```
        q ~ dbeta(5,1)
        # LIKELIHOOD:
        # Estimation of probability of detection
        team1 ~ dbin(q, x)         # seen by team1
        team2 ~ dbin(q * (1-q), x) # missed by team1, seen by team2

        # Step 1. (N = number of plots at each site)
        for(i in 1:N) {
                z[i] ~ dbern(psi[zone.ID.ALL[i]])
                n[i] ~ dpois(lambda[zone.ID.ALL[i]] * a[i] * t[i] * z[i])
                y[i] ~ dbin(q, n[i])
        }
        # Step 2. (M = Study site in chronological survey order)
        for(M in 1:7) {
                psi[M] ~ dunif(0,1)
                lambda[M] ~ dunif(0,4)
                D.hat.ALL[M] <- lambda[M] * psi[M]
                d.hat.ALL[M] <- D.hat.ALL [M] / 0.8500  # p.hat_r.hat <- 0.8500
                mu.hat.ALL[M] <- d.hat.ALL[M] * Area.ALL[M]
        }
} ",fill = TRUE)
sink()
```

Before running jags, bundle the information from the data into: JAGSdata.ALL for compilation of vectors used by the model; params.ALL for a list of parameters of interest; and inits.ALL for the function to create initial values for the model.

```
JAGSdata.ALL <- with(nests.Data.ALL, list(z = any.sign.ALL,
        a = plot.size.ALL, zone.ID.ALL = zone.ID.ALL, y = nests.obs.ALL,
        Area.ALL = Area.ALL, t = period.ALL, N = nrow(nests.Data.ALL),
        team1 = 35, team2 = 5))
params.ALL <- c("psi", "lambda", "D.hat.ALL", "d.hat.ALL", "mu.hat.ALL",
        "q", "x0")
inits.ALL <- function() {list(lambda = rep(1, 7),
        n = nests.Data.ALL$nests.obs.ALL, x0 = 42, q = 0.9)}
```

Save the result as JAGSout.ALL after running jags with the specified Markov chain Monte Carlo (MCMC) settings.

```
# MCMC settings:
ni <- 40000
nt <- 2
nb <- 5000
nc <- 3

# JAGS output:
JAGSout.ALL <- jags(JAGSdata.ALL, inits.ALL, params.ALL,
        "model.txt", n.iter = ni, n.thin = nt, n.burnin = nb, n.chains = nc)
```

Convert the MCMC output into Bwiqid class using the function as.Bwiqid. This Bayesian function converts different classes generated using WinBUGS, OpenBUGS or JAGS into a common class for printing and plotting using wiqid. The results for D.hat ($\hat{D}$), d.hat ($\hat{d}$), lambda ($\hat{\lambda}$), mu.hat ($\hat{\mu}$), and psi ($\hat{\psi}$) were numbered in chronological order of surveys at the seven study sites.

```
attach.jags(JAGSout.ALL)
out.ALL <- as.Bwiqid(JAGSout.ALL)
```

```
Model fitted in JAGS with R2jags
52500 simulations saved.
                  mean       sd   median   HDIlo    HDIup  Rhat n.eff
D.hat.ALL1      1.1942  0.37382   1.1727  0.4624   1.9198 1.001 25000
D.hat.ALL2      1.5206  0.46010   1.5010  0.6326   2.4363 1.001 16000
D.hat.ALL3      0.3979  0.44065   0.2414  0.0000   1.3342 1.001 31000
D.hat.ALL4      0.1461  0.10052   0.1233  0.0068   0.3412 1.001 52000
D.hat.ALL5      0.8648  0.29218   0.8413  0.3130   1.4402 1.001 24000
D.hat.ALL6      0.7008  0.18764   0.6843  0.3444   1.0671 1.001  5900
D.hat.ALL7      0.1932  0.09799   0.1757  0.0369   0.3885 1.001 12000
d.hat.ALL1      1.4050  0.43979   1.3797  0.5440   2.2586 1.001 25000
d.hat.ALL2      1.7890  0.54130   1.7659  0.7442   2.8663 1.001 16000
d.hat.ALL3      0.4681  0.51841   0.2841  0.0000   1.5696 1.001 31000
d.hat.ALL4      0.1719  0.11826   0.1450  0.0080   0.4014 1.001 52000
d.hat.ALL5      1.0174  0.34375   0.9898  0.3682   1.6944 1.001 24000
d.hat.ALL6      0.8245  0.22075   0.8051  0.4052   1.2554 1.001  5900
d.hat.ALL7      0.2273  0.11529   0.2067  0.0436   0.4573 1.001 12000
deviance       79.4797 13.21313  78.6551 54.5287 105.9412 1.001  7500
lambda1         1.4905  0.34798   1.4559  0.8585   2.1889 1.001 17000
lambda2         1.9008  0.41771   1.8600  1.1421   2.7499 1.001  7900
lambda3         1.9988  1.15450   1.9997  0.0148   3.8142 1.001 52000
lambda4         0.2559  0.15097   0.2271  0.0273   0.5552 1.001 52000
lambda5         1.0814  0.28648   1.0501  0.5653   1.6554 1.001 18000
lambda6         0.8009  0.18754   0.7822  0.4636   1.1763 1.001  4600
lambda7         0.3082  0.13051   0.2893  0.0909   0.5735 1.001 12000
mu.hat.ALL1    81.8818 25.63090  80.4077 31.7023 131.6307 1.001 25000
mu.hat.ALL2    40.2157 12.16834  39.6974 16.7306  64.4334 1.001 16000
mu.hat.ALL3    32.4878 35.97737  19.7137  0.0000 108.9326 1.001 31000
mu.hat.ALL4    16.5699 11.40163  13.9802  0.7677  38.6970 1.001 52000
mu.hat.ALL5    46.6369 15.75731  45.3705 16.8790  77.6713 1.001 24000
mu.hat.ALL6   115.4335 30.90530 112.7106 56.7279 175.7626 1.001  5900
mu.hat.ALL7    56.3200 28.56812  51.2117 10.7618 113.2731 1.001 12000
psi1            0.8011  0.16271   0.8418  0.4765   1.0000 1.001 52000
psi2            0.8003  0.16360   0.8415  0.4743   1.0000 1.001 52000
psi3            0.1995  0.16343   0.1583  0.0000   0.5269 1.001 25000
psi4            0.5715  0.17582   0.5795  0.2403   0.9007 1.001 46000
psi5            0.8000  0.16354   0.8419  0.4699   1.0000 1.001 52000
psi6            0.8750  0.11051   0.9058  0.6497   1.0000 1.001 52000
psi7            0.6265  0.16042   0.6370  0.3250   0.9267 1.001 36000
q               0.8133  0.07534   0.8242  0.6588   0.9413 1.002  3600
x0             43.4237  4.60824  42.6675 36.0003  52.6110 1.001  4100

'HDIlo' and 'HDIup' are the limits of a 95% HDI credible interval.
'Rhat' is the potential scale reduction factor (at convergence, Rhat=1).
'n.eff' is a crude measure of effective sample size.
```

Objective #2: To generate histograms of the posterior outputs.

2.1. Estimated orang-utan population (mu.hat)

Run the probability density function for mu.hat at each study site using the `plot` function. Histogram for each study site is generated by changing "mu.hat.ALL1" into "mu.hat.ALL2" ... up to "mu.hat.ALL7".

```
plot(out.ALL, "mu.hat.ALL1", showCurve=FALSE, shadeHDI="skyblue", xlim=c(0,280),
        ylim=c(0,0.048))
```

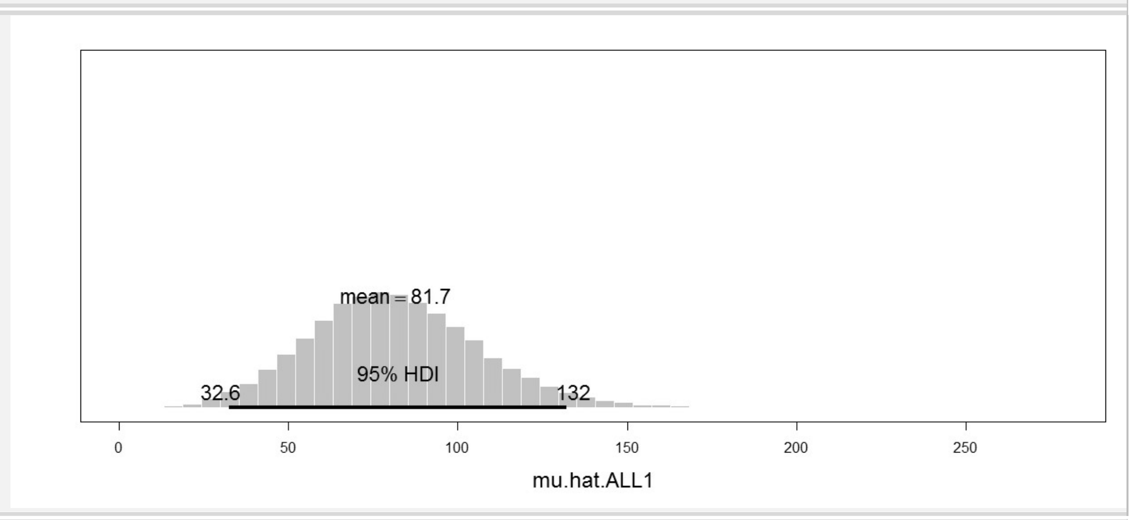

2.2. <u>Estimated orang-utan population (mu.hat) and comparison of percentage overlap between the posterior and prior distributions.</u>

Use the function `postPriorOverlap` to check the percentage of posterior-prior overlap for each site. To compare other study sites, change “mu.hat.ALL[,1]” into “mu.hat.ALL[,2]”… up to “mu.hat.ALL[,7]”.

```
# Step 3. Compare posterior-prior percentage overlap
JAGSdata.ALL$y <- NULL
JAGSout.ALL0 <- jags(JAGSdata.ALL, inits.ALL, params.ALL,
                     "model.txt",n.iter = ni, n.thin = nt, n.burnin = nb,
                     n.chains = nc)
attach.jags(JAGSout.ALL)
postPriorOverlap(mu.hat.ALL[,1],
JAGSout.ALL0$BUGSoutput$sims.list$mu.hat.ALL[,1])
```

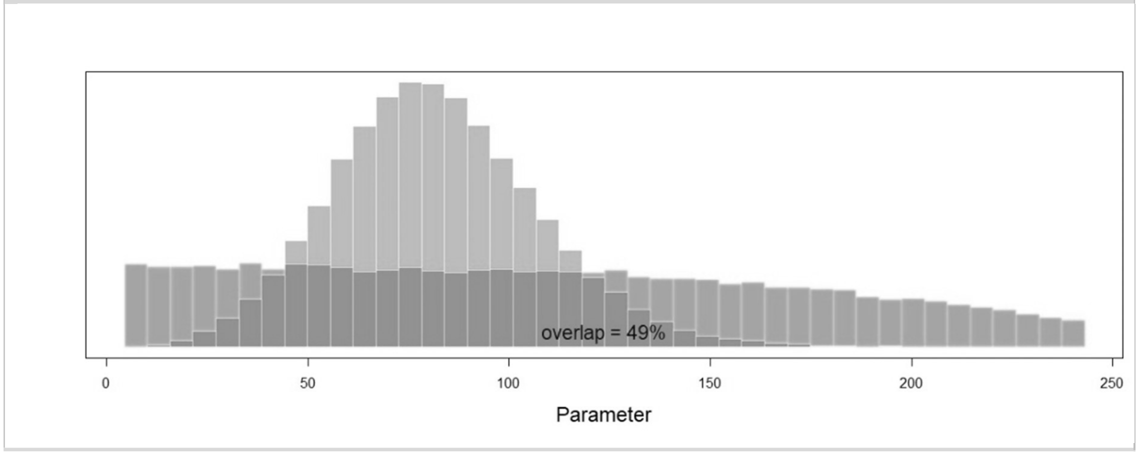

2.3. <u>Estimated probability of old nest at a site (psi).</u>

Generate the probability density function for each study site by changing “psi1” into “psi2” ... up to “psi7”.

```
plot(out.ALL, "psi1", showCurve=FALSE, shadeHDI="skyblue", xlim=c(0,1),
        ylim=c(0,7))
```

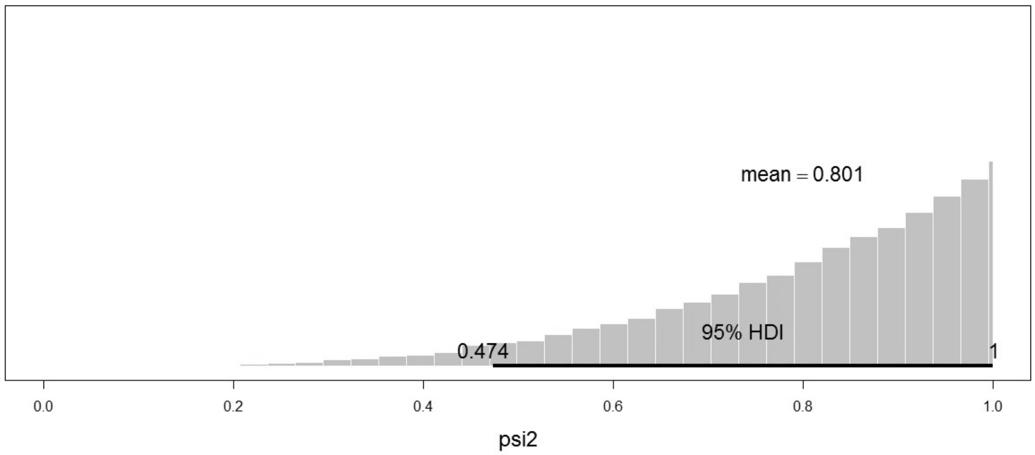

2.4. Estimated probability of detecting new nest by the two teams on the first survey (*q*) with 95% HDI

Run the probability density function for *q* for all seven study sites using the `plot` function.

```
plot(out.ALL, "q", showCurve=FALSE, shadeHDI="skyblue", xlim=c(0,1),
        ylim=c(0,6))
```

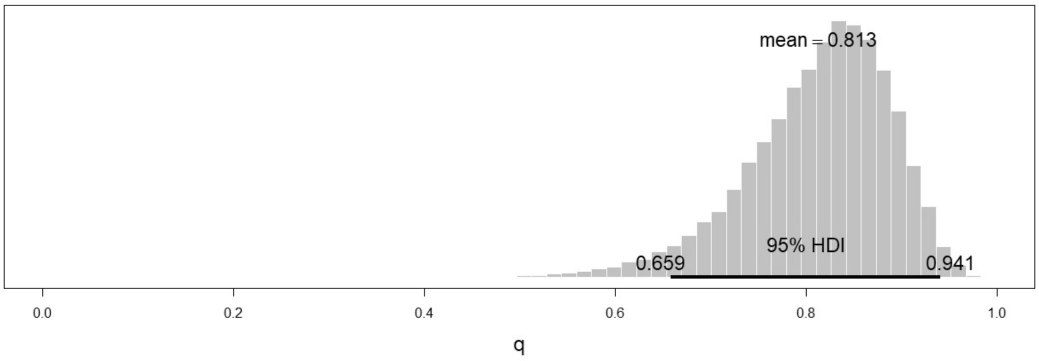

References for Appendices S1 to S5

1 Ancrenaz, M., Goossens, B., Gimenez, O., Sawang, A. & Lackman-Ancrenaz, I. Determination of ape distribution and population size using ground and aerial surveys: a case study with orang-utans in lower Kinabatangan, Sabah, Malaysia. *Animal Conservation* **7**, 375-385, doi:10.1017/s136794300400157x (2004).

2 Bennett, E. L. *Report On A Survey Of Primates In The Maludam Area*. (Wildlife Conservation Society, 1994).

3 Bennett, E. L. *Report On Aerial And Ground Surveys Of The Proposed Maludam National Park: Illegal Logging And Its Implications For The Proposed Park*. (Wildlife Conservation Society, 1999).

4 Gumal, M., Sompud, J. & Kong, D. *Wildlife Survey Of The Proposed Extension To Maludam National Park, Betong Division, Sarawak*. (Wildlife Conservation Society (WCS)-Malaysia Program and Forest Department Sarawak, 2004).

5 Hon, J. & Gumal, M. *Monitoring And Conserving Primates In Maludam National Park, Betong Division, Sarawak*. (Wildlife Conservation Society (WCS)-Malaysia Program and Forest Department Sarawak, 2004).

6 Pearce, K. G. *Development of Lanjak-Entimau Wildlife Sanctuary as a Totally Protected Area Phase IV: The Lanjak-Entimau Wildlife Sanctuary Strategic Management Plan (2009-2020)*. (ITTO-Forest Department Sarawak, 2010).

7 Meredith, M. E. A faunal survey of Batang Ai National Park, Sarawak, Malaysia. *Sarawak Mus. J.* **48**, 133-155 (1995).

8 Gumal, M. *et al.* Densities of orangutan nests in Batang Ai National Park, Lanjak-Entimau Wildlife Sanctuary and the proposed Ulu Sebuyau National Park. *Hornbill* **9**, 177-200 (2007).

9 Gumal, M., Ng, S., Teh, L., Ngelai, M. & Pandong, J. *Ulu Sungai Menyang: Orang-utan Strategic Action Plan*. (in prep).

10 Wich, S. A. & Boyko, R. H. Which factors determine orangutan nests' detection probability along transects? *Trop. Conserv. Sci.* **4**, 53-63 (2011).

11 van Schaik, C. P., Priatna, A. & Priatna, D. in *The Neglected Ape* (eds Ronald D. Nadler, Birute F. M. Galdikas, Lori K. Sheeran, & Norm Rosen) 129-147 (Plenum Press, 1995).

12 Kéry, M. & Royle, J. A. in *Applied Hierarchical Modeling in Ecology: Analysis of distribution, abundance and species richness in R and BUGS* Vol. 1 Ch. 6.Modeling abundance with counts of unmarked individuals in closed populations: Binomial N-mixture models, 219-312 (Academic Press, 2016).

13 Gimenez, O., Morgan, B., J.T. & Brooks, S. P. in *Modeling Demographic Processes in Marked Populations Environmental and Ecological Statistics* (eds David L. Thomson, Evan G. Cooch, & Michael J. Conroy) Ch. Weak identifiability in models for mark-recapture-recovery data, (Springer US, 2009).

14 Plummer, M. JAGS: A program for analysis of Bayesian Graphical Models using Gibbs Sampling. *3rd International Workshop on Distributed Statistical Computing (DSC)* (2003).

15 R Core Team. R: A language and environment for statistical computing. R Foundation for Statistical Computing. (2017). <http://www.R-project.org/>.

16 Su, Y.-S. & Yajima, M. R2jags: Using R to run 'JAGS'. R package version 0.5-7. (2015). <https://CRAN.R-project.org/package=R2jags>.

17 Meredith, M. E. wiqid: Quick and Dirty Estimates for Wildlife Populations. R package version 0.1.3. (2017). <https://CRAN.R-project.org/package=wiqid>.
